# From CBCT to MR-Linac in Image-Guided Prostate Cancer Radiotherapy Towards Treatment Personalization

**DOI:** 10.3390/curroncol32060291

**Published:** 2025-05-22

**Authors:** Florentina Larisa Coc, Loredana G. Marcu

**Affiliations:** 1 Faculty of Physics, West University of Timisoara, 300223 Timisoara, Romania; larisa.coc@e-uvt.ro; 2 Bihor County Emergency Clinical Hospital, 410167 Oradea, Romania; 3 Faculty of Informatics & Science, University of Oradea, 410087 Oradea, Romania; 4 Allied Health & Human Performance, University of South Australia, Adelaide, SA 5001, Australia

**Keywords:** image-guided radiotherapy, geometric errors, PTV margin, organs at risk, adaptive radiotherapy, tumor control, quality of life

## Abstract

**Purpose**: Image-guided radiotherapy (IGRT) has been widely implemented in the treatment of prostate cancer, offering a number of advantages regarding the precision of dose delivery. This study provides an overview of factors, clinical and physical alike, that increase treatment accuracy in prostate cancer radiotherapy in the context of IGRT. The following aspects are explored based on recent literature: the radiotherapy technique used in conjunction with IGRT, the type and frequency of IGRT, the impact of radiotherapy technique/IGRT on target dosimetry and organs at risk, the influence of IGRT on planning target volume margins, the impact of treatment time on dosimetric outcome and clinical outcomes using IGRT repositioning or an online adaptive plan. **Methods**: A systematic search of the literature was conducted within Pubmed/Medline databases to find relevant studies. Of the 152 articles fulfilling the initial search criteria, 79 were selected for final analysis. **Results**: The frequency of image guidance, the treatment regimen and the radiation technique are important factors that contribute to the optimization and personalization of the treatment plan. The daily anatomy and volume of the bladder and rectum can vary considerably, which can significantly impact the dosimetric effects on these organs. When used in conjunction with volumetric modulated arc therapy, IGRT allows for shaping the dose distribution to avoid nearby critical structures such as the bladder and rectum. **Conclusions**: Precise tumor targeting via IGRT can result in fewer geometric uncertainties, thereby improving treatment outcome both in terms of superior target coverage and sparing organs at risk.

## 1. Introduction

According to the latest statistics, prostate cancer (PCa) is the second most common cancer in men in Western Europe and the United States and the fourth most common cancer overall [1]. External beam radiotherapy (EBRT) is the main treatment option for many patients diagnosed with PCa owing to increased efficiency compared to other treatment techniques [2]. In recent years, radiotherapy has undergone significant advancements, enabling the delivery of a more precise and conformal dose to the prostate while simultaneously reducing the dose to surrounding organs at risk (OARs) [3]. In view of this, three-dimensional conformal radiation therapy (3D CRT) has been replaced by techniques employing intensity-modulated beams, such as IMRT (intensity-modulated radiotherapy) or VMAT (volumetric modulated arc therapy) [4]. Yet, compared to 3D CRT, IMRT has some acknowledged disadvantages, such as increased monitor units (MUs) and longer treatment delivery time (even double), which can result in higher positioning errors [5]. For instance, if the treatment time were to be doubled from 1 to 2 min, software analysis suggests that the prostate may move between 750 and 1500 times over the treatment course [6]. VMAT was shown to overcome these drawbacks through the use of arcs or semi-arcs to deliver the treatment in a continuous rotation of the gantry [5].

Both IMRT and VMAT require high accuracy in tumor localization due to possible variations in patient positioning caused by setup errors. Image-guided radiotherapy (IGRT) techniques, when used in conjunction with IMRT or VMAT, can significantly improve treatment accuracy. IGRT techniques include two-dimensional megavoltage (MV) or kilovoltage (kV) imaging, cone-beam computed tomography (CBCT) and megavoltage computed tomography (MVCT). Additionally, non-radiative techniques, such as ultrasound, the Calypso system, the AlignRT system or magnetic resonance imaging (MRI), can be employed to monitor the position of the prostate in real time. One of the key roles of IGRT in prostate cancer radiotherapy is to increase treatment precision and to reduce the planning target volume (PTV) margins for better conformity and organ sparing [2].

While radiotherapy delivery aims for high precision, there are factors that can limit accuracy. It is important to note that errors can occur despite all efforts to minimize them. These errors are the result of a number of factors, such as tumor motion, weight loss, and changes in patient posture or organ volumes. In addition to interfractional variations, it is essential to consider intrafractional variations caused by internal physiological movements such as breathing, rectal gas, swallowing or heartbeat. To ensure accurate treatment, it is necessary to (re)optimize the treatment plan with appropriate PTV margins, taking into account the movement of the prostate. Differences in PTV margins may occur due to variations in the frequency of IGRT techniques, time intervals between IGRT and treatment, and differences in bladder and rectal filling from one radiotherapy session to the next [7].

Adaptive radiotherapy (ART) is a solution that adjusts treatment plans according to these changes. ART can be conducted in two ways: online, with adjustments made during treatment sessions, or offline, with adjustments made between sessions [8]. The MR-Linac has numerous advantages, including improved anatomic visualization of OARs, being a potential treatment approach for reirradiation of prostate cancer after intraprostatic recurrence while also offering the option for direct soft-tissue-based gating [9]. However, MR images are prone to geometric distortions when treating patients with metal prostheses/implants [10], and treatment time may be prolonged (up to 45–60 min) [11].

The purpose of this study is to provide an overview of factors contributing to treatment precision and personalization in prostate cancer radiotherapy in the context of IGRT. To fulfil this aim, the following aspects were investigated based on the published literature: EBRT used in combination with IGRT; the IGRT method and frequency; the impact of technique/IGRT on target and OAR dosimetry, including reoptimization or adaptive radiotherapy; the decision on PTV margins according to IGRT indications; and the effect of treatment time on dosimetric and clinical outcomes.

## 2. Methods—Literature Search

This systematic review is reported according to the 2020 PRISMA (Preferred Reporting Items for Systematic Review and Meta-Analysis) guidelines, and it has been registered with the Open Science Framework with the registration code OSF.IO/342MR being available at: https://osf.io/rmavj (accessed on 12 April 2025).

**Figure 1:**
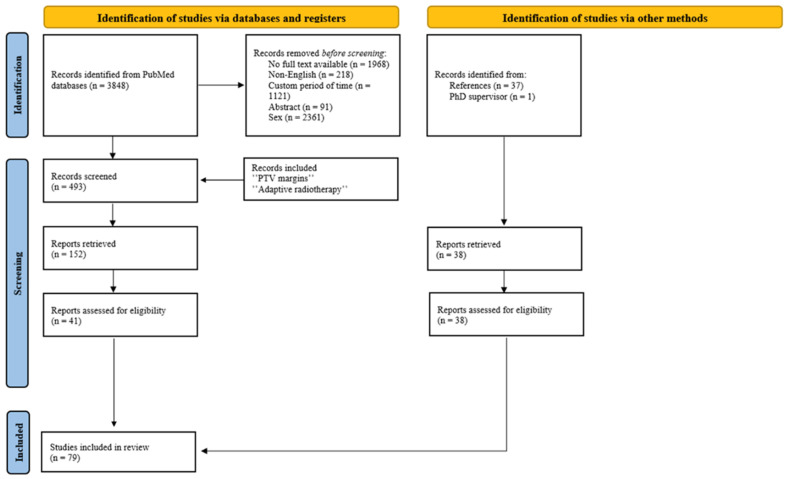
PRISMA flow diagram for literature search.

The search of the literature was conducted within the Pubmed/Medline databases for scientific articles published over the past 10 years (2014–2025) using the following as keywords: “prostate cancer” AND “intensity modulated radiotherapy OR IMRT OR volumetric modulated radiotherapy OR VMAT” AND “image-guided radiation therapy OR IGRT”. Studies were included regardless of tumor stage or treatment status, as long as the patients received external radiotherapy. The present study exclusively considers full-text articles that focus on male patients and are written in English. Reviews, abstracts and conference proceedings were excluded. As a result, 493 articles met the search criteria. The search was narrowed down to articles focusing on treatment personalization using the keywords “PTV margins” and “adaptive radiotherapy”. Article filtration was thus facilitated, resulting in the identification of 152 publications (74 pertaining to PTV margins and 78 to adaptive radiotherapy). Articles concerning fewer than 10 patients or non-EBRT (brachytherapy), as well as articles that provide only radiation effects on hematological parameters or those not presenting a quantitative dose analysis, were excluded, leading to 79 eligible articles (Figure 1).

## 3. Results

### 3.1. Imaging Techniques Used on Board the Linear Accelerator to Guide Prostate Cancer Treatment

**Figure 2:**
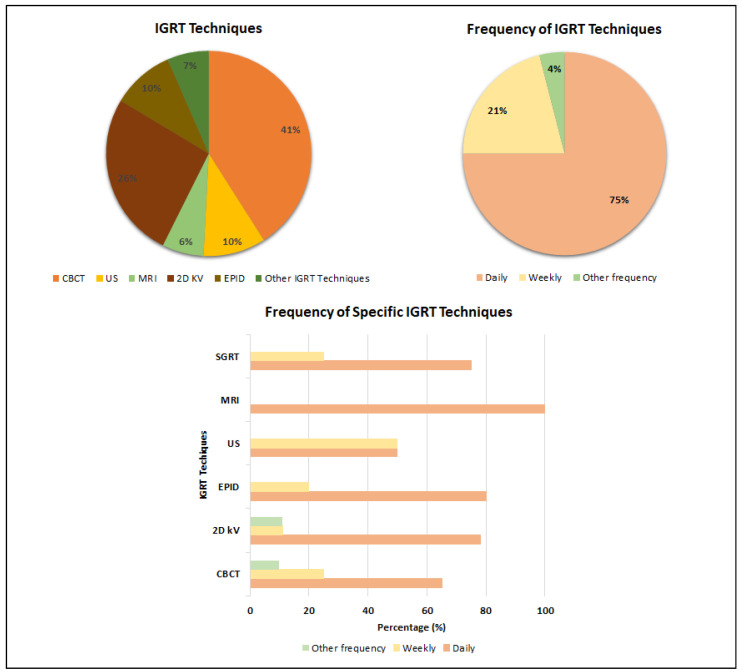
Most commonly used IGRT techniques and their frequency in prostate cancer radiotherapy.

To increase treatment precision delivery, modern treatment techniques have incorporated pretreatment imaging to account for, prevent and/or correct any patient or organ movement that may occur. Several IGRT techniques are implemented in clinics, and the most common ones, together with their prevalence in prostate cancer radiotherapy, are shown in Figure 2.

Based on the last 10 years’ statistics, **CBCT** is the most commonly employed imaging method in prostate radiotherapy, with 41% of the analyzed articles reporting its regular use. CBCT provides better visualization of the bladder and rectum, though at the expense of higher imaging dose, particularly for MV systems (EPID MV 30–70 mGy/image, EPID kV 1–3 mGy/image, CBCT 30–50 mGy/image), and a twofold acquisition time [12].

As shown in Figure 2, another commonly used IGRT method applied in 26% of cases reported in the literature is two-dimensional **orthogonal kV imaging with or without fiducial markers** (FM). FM can facilitate the tracking of inter- or intrafractional movements of the prostate; therefore, FM-based IGRT is an effective tool that can increase the accuracy of radiotherapy while minimizing the radiation dose to normal tissues [13].

**Magnetic resonance imaging (MRI)** has been integrated into linear accelerators to create MR-Linac systems (MRIgRT) to facilitate adaptive treatments and motion management. The primary advantage of this technology is the superior soft-tissue contrast it provides [14], as well as its capacity to control intrafraction motion without the need for additional dose to the patient [15].

**Surface-Guided Radiotherapy (SGRT)** creates 3D images of the patient’s surface, which are compared to a reference surface to assist with positioning. Using SGRT for prostate cancer provided a faster and more accurate patient positioning compared to the conventional three-point localization setup [16].

**Ultrasound imaging** offers an alternative solution for verifying the treatment setting in order to quantify changes in bladder and rectal filling levels, as it is a non-invasive and non-radiative procedure [17].

Figure 2 illustrates **the frequency of IGRT** use in prostate cancer radiotherapy as conveyed from the literature. While most centers reported daily usage of IGRT, around 20% employ image guidance for prostate radiotherapy on a weekly basis. A limited number of clinics reported other IGRT regimens, including the use of IGRT for the first 3 days of treatment, followed by weekly imaging every other day or daily for the first week, followed by once-a-week imaging. The technique, which is used less frequently on a daily basis, is either in the initial stages of implementation or is employed in conjunction with other IGRT techniques.

A limited number of studies evaluated the association between the type/frequency of image guidance and treatment-related adverse effects [18,19]. In their IMRT study, Serizawa et al. compared the outcomes and toxicities of patients treated with IGRT using fiducial markers vs. patients treated without image guidance (non-IGRT). The IGRT cohort was divided into the IGRT-A group, which used the same margins as the non-IGRT group, and the IGRT-B group, with reduced margins. In comparison with non-IGRT, the IGRT-A group showed a lower rate of grade 2+ late GU toxicities (16% vs. 28%), as well as a lower rate of grade 1+ acute GI toxicities (44% vs. 55%). Furthermore, when compared to the IGRT-A group, the IGRT-B group had reduced rates of acute grade 2+ GU (21% vs. 45%) and acute grade 1+ GI toxicities (18% vs. 44%), illustrating the protective role of reduced tumor margins on the adjacent organs at risk [18].

Ghanem et al. compared the impact of daily (arm A) vs. irregular IGRT (arm B), with arm A undergoing IMRT with daily CBCT and arm B patients being positioned on skin tattoos, with 3D-US or 2D KV scans performed on a weekly or biweekly basis. Arm A presented fewer grade 2 (18.1% vs. 28.6%) and grade 3 (1.4% vs. 6.5%) late GU toxicities than arm B, with the most common late GU toxicities being irritative symptoms, hematuria and urethral stricture [19]. The study reiterated the role of frequent (daily) IGRT for accurate targeting, which, inherently, leads to better normal tissue protection.

### 3.2. Positioning Errors in Prostate Cancer Treatment

A patient setup error is defined as a discrepancy between the actual and expected position of the patient relative to the treatment beam, recorded along the X, Y and Z axes, respectively. These errors are then corrected according to each department’s protocol [20]. Setup errors can extend to a six-degree (6D) scope (three translational and three rotational setup errors). Unfortunately, rotational setup errors are not routinely corrected because most currently available couches can only correct one degree of these rotational movements [21]. Motion variation within a fraction is referred to as intrafractional error, while the variation between fractions is designated as interfractional error. These errors have been classified into random (σ) and systematic (Σ), and for each setting, they are calculated based on inter- and intrafractional changes in the prostate. Random errors are statistical fluctuations (alterations in the patient’s position and internal anatomy, such as those resulting from respiration, bladder filling, or rectal distention), while systematic ones are often due to a problem that persists throughout the treatment (difficulties in target delineation due to tumor regression or growth, discrepancies in laser alignment between CT and linear accelerator, or isocenter location) [22,23]. Their timely identification and implementation of counteracting measures are essential [23,24].

Based on the above-mentioned setup verification systems, Table S1 presents positioning errors as reported by the literature in terms of interfractional and intrafractional errors.

#### 3.2.1. Interfractional Error Management in Prostate IGRT

The minimization of interfractional errors can be achieved through the implementation of optimal IGRT, facilitating real-time imaging of the prostate. Alternatively, an increase in the frequency of IGRT can be used to reduce the incidence of inaccuracies.

**Figure 3:**
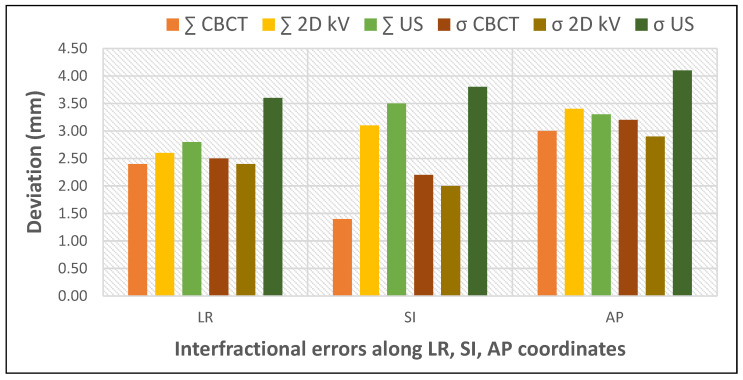
Impact of different IGRT techniques on interfractional errors (based on data from [25]).

To evaluate the advantages and limitations of various IGRT techniques, Mayyas et al. performed a comparative study employing three different IGRT modalities: 3D ultrasound, kV planar imaging and CBCT (Figure 3). The correlation between the shifts evaluated by CBCT and 2D kV was found to be high in the LR and AP directions but low in the SI direction. This may be related to the difficulty in accurately visualizing the prostate apex and base on CBCT. A lower correlation was also observed between 2D kV and US in the AP direction. The time interval between scans (CBCT followed by 2D kV and US, with a minimum of a 10 min delay between the CBCT and US) is a potential explanation for the observed differences, as setup and physiological changes could have occurred between the initial CBCT and the US scan. The interfractional errors were the highest for US (mean error in AP direction −1.2 mm CBCT; −2.9 mm 2D kV; −3.6 mm US). This study also provides insight into the additional dose due to imaging scans throughout treatment: five CBCT scans per week at 2 cGy/scan or five orthogonal image pairs/week at 0.1 cGy/image added approximately 98 cGy additional dose to the treatment [25].

**Figure 4:**
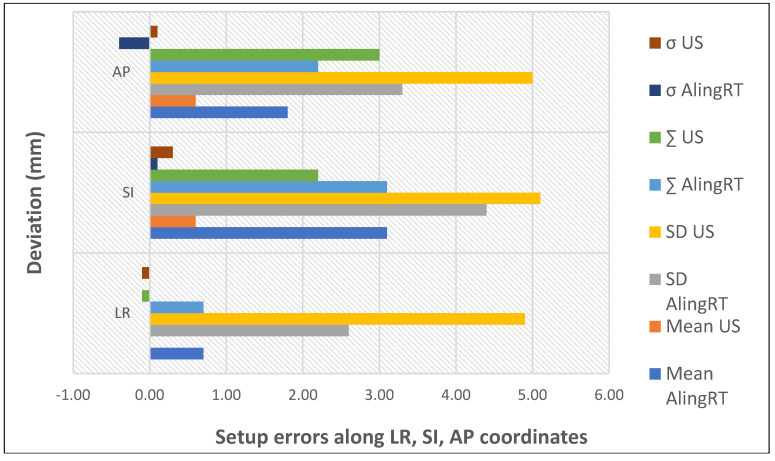
Setup errors quantified by non-invasive IGRT (based on data from [26]).

Krengli et al. compared the daily variations detected by two non-ionizing IGRT techniques, 3D surface imaging and transabdominal ultrasound, to examine the configuration of prostate and internal organ variation (Figure 4). Systematic errors detected by 3D surface and 3D US imaging were significantly different only in the LR direction, which was caused by the difficulty in precisely defining the lateral border of the prostate by ultrasound. Therefore, these two techniques could be used as complementary quality assurance methods and could serve as a daily non-invasive IGRT technique in prostate radiotherapy [26].

Rosenschöld et al. highlighted the importance of IGRT frequency while comparing alternative pretreatment image guidance protocols by using the first fraction weekly and daily. The study demonstrated that weekly 2D kV compared to the initial treatment session resulted in a reduction in systematic errors (from 0.15, 0.26, 0.21 to 0.08, 0.12, 0.09 (mm)), but the random errors remained high (from 0.28, 0.42, 0.29 to 0.35, 0.29, 0.37 (mm) on the lateral, longitudinal and vertical axes, respectively). Additionally, it was observed that in patients with an elevated body mass index, a more rigorous IGRT protocol is necessary, such as daily IGRT. The implementation of regular IGRT in this patient group reduced random errors to 0.1 mm on all three axes [27].

Liu et al. analyzed interfractional organ motion after prostatectomy, evaluating three treatment plans for the same patient: a plan using IGRT repositioning, an online adaptive plan by adapting the original plan to conform to the anatomy of the day and a new plan reoptimized entirely based on the daily anatomy. Data from the study showed that online replanning can correct both systematic and random errors and has the advantage of keeping the original dose distribution throughout the treatment sessions [28].

**Figure 5:**
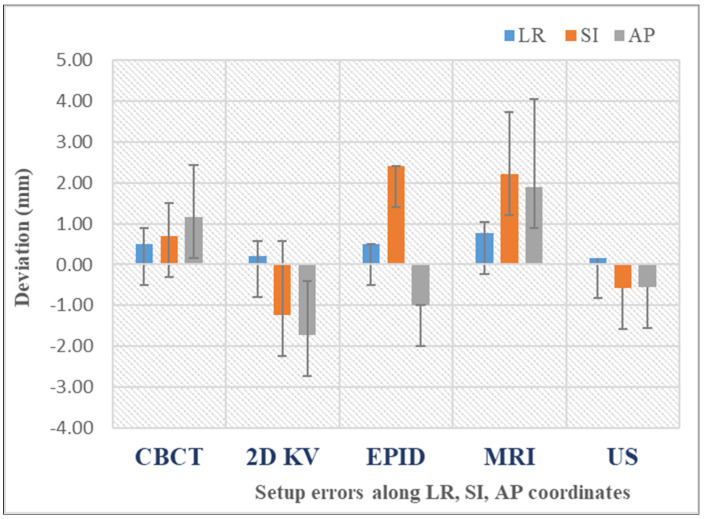
Mean setup errors for various IGRT techniques. The bars represent the standard deviation of the dataset relative to the mean.

Figure 5 illustrates the mean setup errors for the main IGRT techniques used in prostate cancer, as analyzed by this review [13,25,26,29,30,31,32,33,34,35,36,37,38,39]. The mean error is generally used to denote the systematic setup error, which is defined as the average deviation between the planned and actual patient positions across all treatment fractions. The data reveal that CBCT improves positioning precision due to the 3D reference. Planar kV images are quick but only show bony structures, whereas CBCT highlights soft tissues, offering a higher positioning accuracy. Also, while the data show that US imaging can offer similar accuracy to CBCT in pretreatment positioning, generally, it is not safely interchangeable with the latter due to poorer image quality. Recent advancements in MR-Linac technology have significantly increased its use for assessing prostate intrafraction motion. This rise is due to improved soft tissue contrast, continuous imaging without added dose, and the elimination of the need for fiducial markers.

#### 3.2.2. Intrafractional Error Management in Prostate IGRT

Intrafractional movement of the prostate is assessed by considering changes in target position relative to bony anatomy, tracking based on fiducial markers or electromagnetic transponders. From the analysis of the literature and data quantification in Table S1, it can be concluded that intrafractional movement of the prostate is influenced by the degree of bladder filling, rectal diameter and the size and shape of the prostate.

The study conducted by Ballhausen et al. demonstrated that the length of treatment is a significant factor influencing the extent of intrafractional movement of the prostate. The intrafractional motion of the prostate was recorded by real-time four-dimensional ultrasound (4DUS) in 28 patients, 14 being treated with step-and-shoot IMRT, while the other 14 patients were treated with VMAT. The mean prostate radial displacement per fraction was found to be substantially and significantly reduced, from 1.31 ± 1.28 mm (*n* = 357 IMRT fractions) to 0.96 ± 1.04 mm (*n* = 363 VMAT fractions). The prostate remained at 4.55 mm distance from the isocenter for 95% of the fraction time during IMRT and at 2.45 mm distance during VMAT. The variance of the displacements increased linearly with time [40].

For optimal treatment delivery, Tatar et al. recommend MR-guided radiotherapy with daily plan adaptation. They observed that prostate coverage was achieved in 49 out of 50 fractions with a 5 mm PTV without plan adaptation. However, coverage of the seminal vesicles (SVs) was insufficient in 15 out of 50 fractions, which was corrected through adaptive radiotherapy while reducing intrafractional motion [41].

### 3.3. The Influence of Positioning Errors on the PTV Margin

A personalized treatment plan in prostate cancer radiotherapy can only be created with appropriate PTV margins; thus, there are several recommended methods for calculating these margins (see, for example, [42]):

- ICRU Report 62: PTV margin = ∑ + 0.7σ,
- Stroom’s method: PTV margin = 2 ∑ + 0.7σ,
- Van Herk’s formula: PTV margin = 2.5 ∑ + 0.7σ.

In addition to the size of errors, several factors can affect the PTV margin, such as CTV margins, tumor cell density, tumor cell aggressiveness and heterogeneity, as well as the vicinity of normal structures [23]. The PTV margins reported by prostate studies published in the literature are shown in Table S2. In most studies, the PTV margins were calculated based on pretreatment CBCT images using the Van Herk formula.

For example, Su et al. showed that fiducial marker matching results in the smallest PTV margins for the prostate (1.5 + 0.2 mm, 3.5 + 0.5 mm, 2.7 + 0.4 mm in LR, SI and AP direction, respectively), while bone matching leads to the smallest PTV margins for the lymph nodes (0.7 + 0.1 mm, 1.5 + 0.2 mm, 1.4 + 0.2 mm in LR, SI and AP direction, respectively). On the other hand, tattoo matching led to the highest PTV margins, approximately double compared to matching on FM, for both prostate and lymph nodes, the latter being increased due to the interfractional movement of the prostate relative to the pelvic bones [43]. Within the same context, Mayyas et al. reported that each technique (US, kV and CBCT) provided additional information on the reproducibility of the prostate configuration during treatment and reduced the PTV margin by 4 mm compared to alignment on tattoos [25].

Furthermore, Ghaffari et al. reported a set of PTV margins in their daily EPID study (4.0 mm, 3.3 mm and 3.0 mm in the lateral, longitudinal and vertical axes, respectively), which, in the absence of daily verification and correction, would be required to increase (5.4 mm, 5.8 mm and 5.5 mm along the LR, SI and AP directions, respectively) to achieve a 95% CTV coverage [44].

Recent studies indicate that the most effective reduction in PTV margin while maintaining the prescribed dose to the prostate is achieved via MR-Linac due to real-time adjustments in target geometry and volume during adaptive radiotherapy: 65% of patients moved less than 2 mm in any direction during movement monitoring period, while 30% moved 3 mm [45,46].

**Figure 6:**
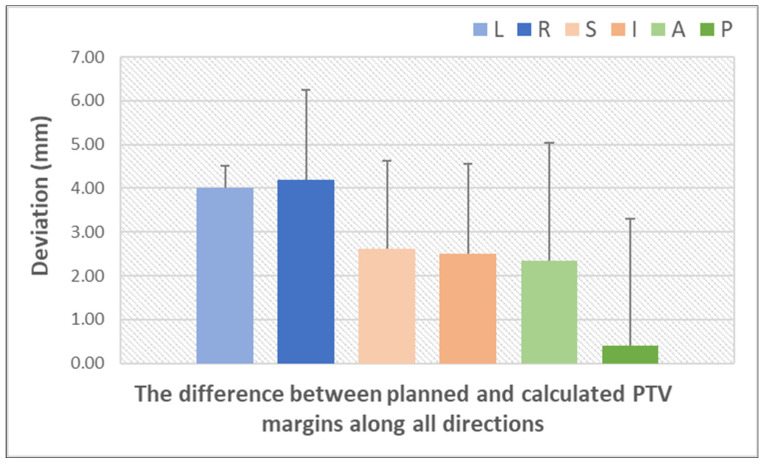
Quantification of setup errors by IGRT techniques (based on data from Table S2) (L = left; R = right; S = superior; I = inferior; A = anterior; P = posterior). The bars represent the standard deviation of the dataset relative to the mean.

Figure 6 illustrates the differences between the PTV margin established in the planning phase and the PTV margin calculated using the van Herk formula, after accounting for systematic and random errors using IGRT [20,29,38,47,48,49,50,51,52]. This graph underscores the significance of IGRT in prostate radiotherapy, demonstrating the way monitoring prostate movement enables the optimization of treatment plans by reducing the margin while maintaining the accuracy of dose delivery to the CTV and minimizing exposure to critical structures. The largest discrepancies between the planning and calculated PTV margins occurred along the LR direction. In contrast, PTV margins along the SI and AP directions resulted in smaller discrepancies. It is plausible that the observed change in the AP and SI directions was due to patient relaxation or a change in bladder and rectal fullness. This also demonstrates the necessity of an anisotropic edge. Treatment margins should be patient-dependent or individualized to maximize the benefit of conformal dose distributions in VMAT and IMRT.

### 3.4. Dosimetric Impact of PTV Margin Optimization on Target Coverage and OARs

The impact of geometric errors and PTV margins on dose distribution, hot and cold spots and tumor volume coverage in prostate cancer radiotherapy are summarized in Supplementary Table S3.

Table S4 summarizes the dosimetric values estimated for the rectum and bladder as the main organs at risk, based on the published literature. Dosimetric parameters for the femoral head and other organs less frequently reported in the literature (such as the sigmoid colon, the penile bulb and the urethra) are also shown in this table.

In the study reported by Li et al., the impact of PTV margin on dosimetric parameters pertaining to the rectum and urinary bladder was observed, as well as the difference between planned and delivered dose. It seems that there is a correlation between the reduction in PTV margins and the decreased exposure of healthy tissue to radiation. This is evidenced by the decrease in the D25 parameter with PTV margin reduction for both the rectum and bladder, which dropped from 52.8% to 41.95% and from 54.07% to 47.70%, respectively. Upon recalculation of the dose distributions on the CBCT images, it became evident that there were notable variations, particularly in the rectum, where D50 increased from 39.20% to 41.11%. Similarly, in the case of the bladder, its fullness appeared to contribute to the observed change, with the dose decreasing from 32.7% to 31.48%. These observations confirm the potential benefit of daily IGRT in prostate cancer radiotherapy [53].

The objective of the study conducted by Heng et al. was to ascertain the impact of bladder and bowel preparation protocols on dosimetric outcome using IMRT planning and CBCT evaluation of the above organs. A total of 12 patients were included in the study, with 6 undergoing the bladder and bowel preparation protocol and the other 6 serving as the control group. The contoured volumes of the bladder and rectum on CBCT images were compared with those on the planning CT. All patients were treated with a total dose of 78 Gy in 39 fractions over a period of 8 weeks. A comparison of patients with and without a bladder preparation protocol revealed that patients without such instructions presented with a greater bladder volume and a higher degree of dose variation. The maximum variation in rectal volume on the day of treatment was found to be up to +96% due to changes in rectal filling. In contrast, the maximum variations in rectal volume with the bowel preparation protocol were less than 25%. The data confirmed that a rectum and bladder preparation protocol can improve treatment accuracy while also leading to reduced side effects and enhanced quality of life [54].

### Plan Adaptation and Its Impact on Setup Errors

Given that IGRT is unable to fully account for patient-specific treatment variations, the strategy of plan adaptation was introduced with the objective of reducing systematic and random errors. Consequently, Böckelmann et al. devised a clinical approach to evaluate setup errors during treatment sessions for each patient. The initial fractions were employed to assess patient positioning by comparing the planning CT with CBCT scans. In the event of a high degree of concordance between CT and CBCT images, indicating minimal displacement of the rectal balloon and anterior rectal wall, PCa patients were not subjected to an adapted treatment plan. When setup errors indicated greater uncertainties (i.e., the rectal balloon and anterior rectal wall were situated outside the delineated rectal volume), a new planning CT scan was conducted, an adapted set of contours was created, and a treatment plan was developed that adhered to the principles of adapted interfractional patient positioning accuracy for both the initial and adapted treatment plans. The displacements in the LR and SI directions were smaller for the adapted plan (1.12 and 1.72 mm for Σ and 4.17 and 3.75 mm for σ) than for the original plan (1.77 and 2.62 mm for Σ and 4.46 and 5.39 mm for σ). With regard to AP motion, the adapted (1.73 mm for Σ and 3.20 for σ) and initial (1.67 mm for Σ and 3.21 for σ) plans were observed to exhibit comparable characteristics. The CTV-PTV margin in the AP direction demonstrated comparable outcomes, with a range of 6–8 mm between the original and adapted plans. Conversely, the LR and SI margins of the adapted plans showed reductions of 2 mm (5–6 mm and 7–8 mm) and 3 mm (7–8 mm and 10–11 mm), respectively, in comparison to the margins derived from the original plans [47].

Mannerberg et al. investigated the role of adaptive radiotherapy in 35 PCa patients based on two MR images (MR1 and MR2) taken 30 min apart using an MR-Linac. Three ultra-hypofractionated VMAT plans were created based on MR1 with three different PTV margins (7 mm, 5 mm and 3 mm), the plans being recalculated using the second set of MR images (MR2). Results showed an increase in bladder volume by an average of 40.9% between the two images. The difference in rectal volume ranged from 10.9% to 38.8%, with the negative differences in rectal volume being caused by gas. The dose to the CTV was reduced by 1.1% with the 7 mm PTV margin. The corresponding values for 5 mm and 3 mm PTV margins were 2.0% and 4.2%, respectively. While the results confirm the role of adaptive treatment in personalized radiotherapy, the authors suggest that, owing to the slow MR-Linac workflow, target underdosage can occur due to anatomical changes during the investigation [16].

### 3.5. The Impact of Treatment Time on Error Reduction

Shorter treatment times in radiotherapy are directly associated with a reduction in intrafractional motion and delivery uncertainties. For instance, a study by Li et al. has shown that hypofractionated VMAT treatment is effective in reducing treatment time, as compared to IMRT. VMAT delivers the dose in 2–4 min, while conventional IMRT treatment takes 3–7 min. Owing to this, a 7 mm isotropic margin (5 mm posterior) was used in the conventional group, while a 5 mm isotropic margin (4 mm posterior) was used in the hypofractionated group. When dose distributions were recalculated using CBCT images, the results showed that VMAT plans offer better PTV D95% and a reduction in most dosimetric parameters pertaining to organs at risk [53]. In addition, Benedeka et al. showed that using VMAT without a flattening filter for the ultra-hypofractionated delivery of radiation reduces treatment time by around 50% (from 2.3 min to 1.01 min), while keeping the dosimetric effect of organ movement under control. There was no deterioration in the quality of the treatment plan in terms of dose volume parameters or delivery plan verification results [24].

In terms of treatment duration and its impact on plan quality, VMAT was found to demonstrate superiority over three other techniques (3D CRT, 5-field IMRT, helical tomotherapy (HT)). VMAT was better at reducing rectal and bladder toxicity and allowed for a higher dose to the target. The highest MUs were obtained with the HT technique, leading to an average treatment delivery time of 4.70 ± 0.84 min as compared to 3D CRT (0.74 ± 0.04 min), IMRT (1.42 ± 0.26 min) and VMAT (0.87 ± 0.06 min) [55].

The technology and design of the latest linear accelerators allow for further optimization of treatment delivery. In view of this, Pokhrel et al. evaluated the performance of the O-ring Halcyon in prostate cancer in terms of quality, delivery efficiency and accuracy. Two types of stereotactic body radiotherapy (SBRT) plans were generated: one with a Halcyon beam of 6MV-FFF (800 MU/min) and the other with a TrueBeam beam of 6MV-FFF (1400 MU/min). Despite the longer beam start times for Halcyon plans (3.2 min, up to 3.8 min) in comparison to TrueBeam VMAT plans (2.1 min, up to 2.5 min), the total treatment times are comparable, with a mean delivery time of 8.20 ± 0.46 min for the Halcyon linac and 9.90 ± 0.19 min for the traditional C-arm linac [56].

## 4. Discussion

The potential of IGRT to enhance the effectiveness of radiotherapy treatment for patients diagnosed with prostate cancer is well understood, provided that the technology is efficiently utilized. In this context, several studies present the advantages and limitations of IGRT techniques.

For instance, an advantage of CBCT consists of the overlay of 3D reference data with the pretreatment acquisition. This is performed either manually or automatically following one of three procedures: bone alignment, soft tissue alignment (ST) or dual alignment. Some studies have shown that the PTV margins based on soft tissue alignment are smaller than those based on bony structure [29,48]. This translates into a lower risk of late rectal toxicity [57]. However, a study reported by Hirose et al. showed the efficiency of 2D-FM parameters in defining PTV margins, as compared to CBCT-ST, to reduce the risk of under-irradiation [49]. Although fiducial markers can be used to indicate the position of the prostate, they are not capable of conveying the shape of the prostate in real time.

Modern radiotherapy centers are in a position to benefit from the high resolution of MRI. Consequently, prostate cancer studies using MRIgRT have reported an isotropic PTV margin reduction to 3 mm, in contrast with 5 mm with CT-guided RT [58]. Other radiotherapy centers have adopted alternative IGRT techniques (such as SGRT or US), which have been shown to be more cost-effective and better suited to meet the specific resources of each department. Some limitations of the SGRT technique include the presence of hair on the patient’s skin surface, which can compromise the accuracy of positioning due to the loss of surface calculation points [59]. Additionally, daily variations in bladder and bowel filling, which are not evident on the patient’s surface, can also introduce challenges. Also, a number of studies have demonstrated that configurations based on user-dependent transperineal ultrasound (TPUS) and user-independent CBCT lead to large geometric differences, especially in the anteroposterior (AP) direction [30,31]. In light of these discrepancies, it was concluded that ultrasound fails to provide an acceptable level of geometric accuracy for prostate localization. Consequently, these techniques are recommended to be employed in conjunction with other IGRT techniques [60]. This could provide a non-invasive, daily option for prostate cancer patients [26].

The use of IGRT and its frequency can influence decisions regarding the reduction of the PTV margin to achieve better OAR protection while still maintaining the prescribed dose within the volume of interest. For instance, Ariyaratne et al. showed that 90% of patients achieved better target coverage with daily compared to weekly CBCT imaging [61].

Advancements in technology have facilitated more precise monitoring of tumor variations. Initially, tattoo positioning was utilized; subsequently, bony marking was employed; and, finally, fiducial markers were introduced for the identification of soft tissues. In the current era, 3D pretreatment information can be obtained, and even real-time tracking of prostate variations is now possible. Consequently, positioning errors in prostate cancer treatment have been significantly minimized. A number of studies demonstrate the advantage of using fiducial markers for position verification, showing smaller systematic errors in seed matching than in bony matching on the lateral and vertical axes [13,43,49,50]. They also confirmed that random errors associated with seed matching are more significant than those associated with bony matching on the same axes. This may be caused by the motion of the pelvic lymph node, which is influenced by bladder filling and small intestine motion [43].

On the other hand, the advantages of high-quality soft tissue contrast for MR-Linac have shown that bone registration, with or without FM, cannot be a perfect surrogate for prostate registration; therefore, online adaptive planning is required for accurate treatment delivery. Nikol et al. used three FMs and observed that the FMs migrated by 0.05 mm/fraction due to prostatic deformation [62], while Kim et al. observed interfractional variability based on bone anatomy and prostate registration, showing a strong correlation only in the AP direction: mean error 0.57, 2.28, 2.45 (mm) after bone localization and 0.76, 2.02, 1.89 (mm) after prostate localization on the lateral and vertical axes [32]. This finding aligns with numerous others that have hypothesized that MR-Linac and online adaptive radiotherapy could facilitate the reduction of prostate motion during treatment, and PTV margins could be safely reduced to 3 mm [41,63,64].

It can be seen from the evidence presented above that each IGRT technique has its own particular advantages and drawbacks. It is thought that the most accurate solution in patient positioning includes a daily MRI, although many centers use a CBCT/2D kV system with fiducial markers. However, if the patient is not eligible for the insertion of fiducial markers, it may be beneficial to consider combining CBCT or 2D kV with non-ionizing IGRT ultrasonography or 3D surface imaging, allowing movement monitoring in real time.

Studies to date suggest that it is difficult, if not impossible, to completely eliminate all geometric errors during prostate radiotherapy. However, daily IGRT was shown to significantly reduce PTV margins [51].

It is to be noted that successfully achieving optimal dosimetry for one criterion, e.g., PTV coverage, is often in conflict with meeting the requirements for the other criterion, i.e., the restrictions imposed by OARs. There are situations when complications are acceptable; in the case of volumetrically applied dose constraints, this means that the risk of complications depends on the dose distributed throughout the organ. In contrast, for serial organs, complications due to geometric errors are unacceptable, and the risk of complications depends strongly on the region receiving the maximum dose [65].

As most studies indicate, the quality of target volume coverage increases as the PTV margin increases. However, increasing the PTV margin has the downside of high toxicity to organs at risk. Therefore, it is necessary to explore other methods to maintain clinically adequate tumor volume coverage while keeping the PTV margin low. For this purpose, many radiotherapy clinics follow a prostate cancer protocol in which the patient is scanned with a full bladder and empty rectum [54]. The movement of the prostate is limited by these two organs at risk to ensure that the tumor volume receives the planned radiation dose.

Despite advances in treatment planning and delivery, the rectum remains a critical organ that raises caution in prostate cancer patients undergoing radiotherapy. Radiation-induced rectal toxicities continue to be a clinical challenge due to the close proximity of the anterior rectal wall to the prostate gland. Physical devices such as endorectal balloons, hydrogel rectal spacers or rectal retractors have been used to increase the distance between the prostate and the rectum [43]. In addition to these devices, IGRT has allowed for a reduction of the PTV margin in the posterior region [66]. The use of anisotropic margins was shown to increase the probability of tumor control while decreasing normal tissue effects.

Da Silva et al. argued that the determination of the PTV margins depends on the pretreatment imaging system used. In their study, comparable plan quality was obtained between CBCT-guided VMAT plans and MRI-guided IMRT plans using the same PTV margin [1]. However, MRI-guided online adaptive radiotherapy allowed for margin reduction, owing to the additional soft tissue information provided by this imaging technique.

Although MRI is superior to CBCT in terms of OAR dosimetry [14], when compared to 2D pretreatment techniques, CBCT remains advantageous in certain instances. Nakamura et al. found that IMRT with soft tissue-matched CBCT allows for a reduction of the PTV margin without compromising tumor control, which successfully reduced acute rectal toxicity compared to IMRT configured on 2D bone landmarks [2]. Furthermore, Maund et al. suggested that the use of IMRT in conjunction with daily CBCT could result in a reduction of the PTV margin from 5 mm to 3 mm without compromising tumor control and is favorable in terms of reducing rectal toxicity. At the same time, they cautioned that a smaller margin presents a challenge due to the interfractional movement of seminal vesicles [67]. These findings highlight the importance of frequent imaging in achieving better treatment accuracy and increased therapeutic index in radiotherapy [31].

Thus, in order to minimize the discrepancy between the planned and delivered doses, it is essential to ensure that the patient follows the protocol for filling the urinary bladder and emptying the rectum, thereby achieving a volume as close as possible to that on the day of the CT simulation. Rectal and urinary bladder variations can be compensated for by an additional margin. However, this approach also has a disadvantage reflected by the increased radiation-induced toxicity, which can often be tackled by adaptive radiotherapy.

This review has identified numerous studies evaluating the accuracy of dose delivery using different IGRT in prostate cancer. A comparison between reports is challenging due to the large number of variables identified among the evaluated studies, such as image acquisition protocol, frequency of IGRT, treatment planning system (TPS) algorithm and the diversity of dosimetric parameters reported in the articles. However, it seems that previous studies have indicated that MR-Linac represents a future-oriented solution for adaptive radiotherapy in prostate cancer.

As for current challenges, the implementation of online adaptive radiotherapy requires considerable investment in capital, equipment and personnel, which represents a substantial barrier to its adoption. For instance, the Elekta Accelerator has developed a new mechanism that enables position adaptation (online plan adaptation is performed based on the new patient’s position and optimized on the pretreatment CT and contours) and shape adaptation (online plan adaptation is performed on the new patient’s anatomy and optimized on the daily MRI and adapted contours). This allows for the visualization of all anatomical changes during the course of radiotherapy, enabling the adaptation of the treatment plan to ensure optimal results [68].

Another critical aspect of online adaptive MRgRT is that it is time-consuming, not only because MRI is inherently slow, but also due to the multi-step adaptation process. The online adaptation can extend the radiotherapy sessions by 30 to 60 min, impacting treatment efficacy [69]. In this context, the use of high-resolution IGRT that allows for automatic alignment, combined with a hypofractionated treatment regimen, was shown to be among the optimal solutions for delivering effective and rapid treatment [16].

A promising direction for adaptive MRgRT involves the use of quantitative MRI-derived biomarkers, which can provide valuable information about treatment response, allowing clinicians to detect tumor microenvironmental changes that could indicate early responses or resistance to treatment [70]. Current trends show that online adaptive MRI-guided radiotherapy is transforming conventional treatment workflows while also integrating advanced AI-assisted processes to boost efficiency and maintain high standards of dosimetric accuracy during treatment delivery [71].

## 5. Conclusions

Image-guided radiotherapy techniques are key tools in enhancing tumor control by identifying and correcting positioning errors in real time. The type of such technology, frequency of use, and the PTV margins, as well as the target position relative to organs at risk, are all critical factors that dictate treatment outcome. Intensity-modulated treatment techniques were shown to offer a more precise and targeted approach to the tumor, minimizing toxicity and providing superior protection of normal tissues compared to conventional techniques. Furthermore, patients’ compliance regarding prostate cancer treatment protocols (bladder/rectal filling) has an important impact on internal organ movement and should be strictly monitored on a daily basis during the course of radiotherapy. This, in turn, has the potential to enhance the patient’s quality of life.

The role of CBCT in the precise delivery of prostate cancer radiotherapy has been proven by several studies, being considered a key component of the treatment process and an important factor leading to a more personalized treatment approach. It is evident that MRI offers a number of advantages; however, this technology remains accessible to a limited number of centers due to the significant financial investment required. Therefore, most centers use CBCT in conjunction with other IGRT systems (e.g., ultrasound or surface-guided radiotherapy) to increase the precision of prostate cancer radiotherapy.

A summary of the main findings based on the analyzed literature is collated below:

- CBCT is the most frequently employed IGRT technique, representing 41% of pretreatment imaging in prostate cancer radiotherapy.
- Daily IGRT verification improves target volume coverage in 90% of patients compared with weekly imaging.
- To avoid the risk of underdosing the tumor volume, the PTV margin must be kept at 3 mm or above, especially in situations when IGRT is not used daily.
- When patient positioning is based on skin tattoo vs. IGRT, it is recommended for the PTV margin to be doubled.
- As confirmed by a number of studies, VMAT has the advantage of reducing the intrafractional displacement variation of the prostate by half when compared to IMRT.
- Among linac-based techniques, VMAT provides optimal-quality treatment plans, offering a reduction of up to 50% in monitor units and treatment time while achieving the most conformal isodoses.
- MRI-guided radiotherapy represents the next solution for individualized and adaptive treatment in prostate cancer patients.

## Supplementary Materials

The following supporting information can be downloaded at: https://www.mdpi.com/article/10.3390/curroncol32060291/s1, Table S1. Types of interfractional and intrafractional errors and their identification by IGRT in prostate radiotherapy (studies are listed in chronological order) [13,20,22,25,26,27,29,30,31,32,33,34,35,36,37,38,39,43,44,47,48,49,50,51,52,72,73,74,75,76,77,78,79]; Table S2. PTV margin reduction in prostate cancer treatment according to the literature [20,22,25,29,32,33,35,37,38,39,43,44,47,48,49,50,51,52,72,78,79,80] Table S3. PTV-related dosimetric parameters based on the literature (studies are listed in chronological order) [1,5,16,28,51,52,53,55,56,61,64,66,77,80,81,82,83,84,85,86,87,88,89,90,91,92]; Table S4. Dosimetric parameters evaluated for the OARs during prostate cancer radiotherapy (studies are listed in chronological order) [2,4,5,16,28,34,41,53,55,56,61,64,66,77,80,81,82,83,84,85,86,87,88,89,90,91,93,94,95,96,97].
