# Supplementary material for: From CBCT to MR-Linac in Image-Guided Prostate Cancer Radiotherapy Towards Treatment Personalization
Source: Curr Oncol. 2025 May 22;32(6):291. doi: 10.3390/curroncol32060291 (PMC12191943; doi:10.3390/curroncol32060291)
Supplement: Supplementary file 1 [file curroncol-32-00291-s001.zip › Table S1.pdf]

Table S1. Types of interfractional and intrafractional errors and their identification by IGRT in prostate radiotherapy (studies are listed in chronological order)

Technique used / study (ref)	Interfractional errors (mm)			
	Type of error	LR	SI	AP
CBCT				
Adamczyk et al. (2014) [64]	Mean	-0.2	0.1	-2.0
	Median	0.00	0.00	0.00
	SD	2.1	1.3	5.5
	∑	1.0	0.4	3.6
	σ	2.0	1.3	4.6
Mayyas et al. (2014) [25]	$\sum_{daily}$	2.4	1.4	3.0
	σ_{daily}	2.5	2.2	3.2
	Mean_{daily}	1.1	0.2	-1.2
Hirose et al. (2014) [65]	$\text{Mean}_{2\text{ nd fraction}}$	0.1	-0.1	-0.5
	$\text{SD}_{2\text{ nd fraction}}$	1	0.5	3
	$\sum_{2\text{ nd fraction}}$	0.8	0.4	2.4
	$\sigma_{2\text{ nd fraction}}$	0.7	0.3	1.9
	$\text{Mean}_{\text{fracția 3 rd}}$	0.1	-0.1	-0.2
	$\text{SD}_{3\text{ rd fraction}}$	0.9	0.4	2.6
	$\sum_{3\text{ rd fraction}}$	0.6	0.3	1.8
	$\sigma_{3\text{ rd fraction}}$	0.7	0.3	2
	$\text{Mean}_{4\text{ th fraction}}$	0.0	-0.1	-0.3
	$\text{SD}_{4\text{ th fraction}}$	0.9	0.4	2.6
	$\sum_{4\text{ th fraction}}$	0.6	0.3	1.8
	$\sigma_{4\text{ th fraction}}$	0.7	0.3	2
Oehler et al. (2014) [22]	$\sum_{bone}$	0.61	1.72	1.92
	σ_{bone}	0.90	1.69	1.87
	$\sum_{\text{endorectal balloon}}$		1.2	1.68
	$\sigma_{\text{endorectal balloon}}$		1.66	1.82
	$\sum_{FM}$	0.49	0.36	0.77
	σ_{FM}	0.93	0.17	1.09

Shiraishi et al. (2014) [66]	∑	0.67	1.22	1.38
	σ	0.66	1.85	1.35
	Mean	0.28	0.49	0.79
Chiesa et al. (2015) [67]	∑	0.2	0.32	0.19
	σ	0.29	0.3	0.37
	SD	0.5 ± 3.6	-1.4 ± 4.2	-1.4 ± 4.1
Fargier-Voiron et al. (2015) [48]	Mean ± SD CBCT	0.5 ± 3.3	−0.9 ± 4.2	2.8 ± 4.1
	Mean ± SD CBCT residual	0.3 ± 3.0	−1.0 ± 4.2	−0.5 ± 3.9
Sato et al. (2015) [41]	∑	0.4	1.7	2.2
	σ	0.6	1.5	1.8
	Mean ± SD	0.1±0.7	0.9 ±2.2	0.9±2.8
Drozdz et al. (2016) [51]	∑ bone, daily	1.72	1.30	2.38
	σ bone, daily	1.99	1.46	2.33
	∑ FM, daily	1.76	2.09	2.51
	σ FM daily	2.23	2.26	3.20
	∑ 1 st fraction + weekly	2.05	2.29	3.22
	σ 1 st fraction + weekly	2.27	3.35	3.31
	∑ 1 st fraction + every other day	1.24	1.43	197
	σ 1 st fraction + every other day	1.93	2.12	2.89
	∑ nonIGRT	2.56	2.87	3.99
	σ nonIGRT	2.28	2.38	3.31
	∑ debut 3 fractions+ weekly	1.37	1.68	2.04
	σ debut 3 fractions+ weekly	2.23	2.26	3.20
	∑ debut 3 fractions+ every other day	0.84	1.08	1.23
	σ debut 3 fractions+ every other day	1.72	1.87	2.51

	∑ debut 5 fractions+ weekly	1.35	1.18	2.10
	σ debut 5 fractions+ weekly	2.01	2.07	2.95
	∑ debut 5 fractions+ every other day	0.80	0.74	1.26
	σ debut 5 fractions+ every other day	1.67	1.68	2.49
Kanakavelu et al. (2016) [20]	∑	1.61	1.48	1.47
	σ	1.41	1.21	1.19
Jeong et al. (2016) [68]	∑	2.21	1.05	-0.67
	σ	1.95	1.01	1.02
Richte et al. (2016) [47]	Mean ± SD	1.1 ± 3.6	0.3 ± 3.3	0.9 ± 3.2
	Median	1.4	1.2	0.6
Goff et al. (2017) [13]	Mean	-0.4	-1.8	-3.2
	∑	3.3	3.3	5.2
	σ	3.3	2.4	3.7
Rastogi et al. (2017) [69]	$Mean_{bone}$ ± SD	0.4±1.4	2.1±1.2	3.7±2.1
	$Median_{bone}$	0.3	2.14	3.6
	$Mean_{FM}$ ± SD	1.1±1.7	2.3±1.5	4.1±2.3
	$Median_{FM}$	0.11	2.28	4.09
Hirose et al. (2018) [44] (CBCT post-treatment)	∑ soft tissue	0.39	0.85	0.69
	σ soft tissue	0.76	1.41	1.23
Hirose et al. (2018) [44] (CBCT pretreatment, after 2D kV)	∑ soft tissue	0.22	0.33	0.35
	σ soft tissue	0.44	0.8	0.85
Wang et al. (2018) [70]	Mean ±SD	0.572±2.062	−0.952±4.015	−1.116±2.590
	$Mean_{residual}$ ±SD	−0.149±0.796	−0.294±0.720	−0.545±0.686
	R ± SD	3.114±13.937	1.057±8.748	−1.390±8.205
	$R_{residual}$ ± SD	1.321±7.473	0.767±6.039	0.572±5.804
	∑	0.3±2.0	−1.0±2.4	−0.7±1.1
	σ	1.5	2.6	1.9

	$\sum_{residual}$	0.0±5.0	−2.6±0.3	−0.5±0.5
	$\sigma_{residual}$	0.7	0.7	0.6
Ingrosso et al. (2019) [71]	Mean ± SD $_{FM}$	0.90 ± 0.84	0.00 ± 2.07	-0.80 ± 1.28
Su et al. (2019) [32]	$\sum$	0.7	2.3	2.2
	σ	0.7	1.6	1.9
	$\sum_{bone}$	2.5	4.1	4.2
	σ_{bone}	3.0	3.3	2.5
	$\sum_{FM}$	2.1	4.2	3.5
	σ_{FM}	3.1	3.7	3.3
Böckelmann et al. (2020) [38]	$\sum$	1.12	2.28	1.48
	σ	1.89	3.19	2.1
	SD	2.03	4.0	2.53
Kee Oh et al. (2020) [72]	Mean	−0.6	0.3	−1.0
	$\sum$	1.6	1.1	1.9
	σ	2.8	1.8	2.4
2D kV				
Mayyas et al. (2014) [25]	$\sum$	2.6	3.1	3.4
	σ	2.4	2.0	2.9
	Mean	0.5	-0.4	-2.9
	$\sum_{residual}$	0.7	1.0	1.0
	$\sigma_{residual}$	1.8	2.3	2.5
	Mean $_{residual}$	0.2	0.2	-0.4
Hirose et al. (2014) [65]	Mean	-0.2	0.0	-0.3
	SD	0.7	0.4	2.4
	$\sum$	0.3	0.3	1.4
	σ	0.7	0.3	2
Oehler et al. (2014) [22]	$\sum_{bone}$	0.77	1.39	1.78
	σ_{bone}	0.89	2.30	1.97
	$\sum_{endorectal\ balloon}$	1.70	3.20	1.53
	$\sigma_{endorectal\ balloon}$	1.35	3.15	1.36
	$\sum_{FM}$	0.46	1.16	0.88
	σ_{FM}	0.37	0.46	0.71
	$\sum_{tattoo}$	0.23	0.5	0.28

Rosenschöld et al. (2014) [27]	σ_{tattoo}	0.29	0.44	0.31
	$\sum_{1st\ fraction}$	0.15	0.26	0.21
	$\sigma_{1st\ fraction}$	0.28	0.42	0.29
	$\sum_{weekly}$	0.08	0.12	0.09
	σ_{weekly}	0.35	0.52	0.37
	σ_{daily}	0.1	0.1	0.1
Goff et al. (2017) [13]	Mean	0.3	-3.3	-2.0
	$\sum$	2.6	2.9	4.8
	σ	3.3	2.3	2.8
Hirose et al. (2018) [44]	$\sum_{FM}$	0.22	0.33	0.35
	Σ_{FM}	0.44	0.80	0.85
Su et al. (2019) [32]	$\sum$ residual	0.1	0.4	0.3
	σ residual	0.6	0.7	0.9
Knybel et al. (2022) [73]	REB_{mean}	0.93±0.39		
EPID				
Eminowicz et al. (2014) [74]	SD_{daily}	0.2	0.3	0.3
Rudat et al. (2016) [56]	$\sum_{nonIGRT}$	2.0	2.8	2.7
	$\sigma_{nonIGRT}$	3.6	2.8	3.7
	$\sum_{daily}$	1.1	1.5	1.5
	σ_{daily}	2.7	2.4	2.7
Mahdavi et al. (2017) [75]	Mean	0.5	2.4	-1.0
	$\sum$	2.4	2.0	2.7
	σ	6.4	6.1	5.9
	$Mean_{residual}$	-0.1	0.9	-0.5
	$\sum_{residual}$	1.1	1.4	2.4
	$\sigma_{residual}$	3.8	3.6	3.9
Ghaffari et al. (2019) [33]	$\sum$	0.9	0.7	0.54
	σ	2.3	2.2	2.2
US				
Mayyas et al. (2014) [25]	$\sum$	2.8	3.5	3.3
	σ	3.6	3.8	4.1
	Mean	0.0	-1.4	-3.6
	Mean + SD	0.5 ± 2.9	−2.3 ± 4.6	1.3 ± 5.0

Fargier-Voiron et al. (2015) [48]	Mean + SD residual	0.2 ± 2.7	1.0 ± 4.6	−0.7 ± 4.3
Krengli et al. (2016) [26]	Mean	0.0	0.6	0.6
	SD	4.9	5.1	5.0
	∑	-0.1	2.2	3.0
	σ	-0.1	0.3	0.1
Richter et al. (2016) [47]	Mean ± SD	−0.4 ±3.8	0.8 ± 4.1	−0.5 ± 4.4
	Median	−0.3	1.0	−0.8
OTHER				
**TATTOO** Hirose et al. (2014) [65]	Mean	0.2	0.0	-0.1
	SD	3.1	3.1	3.1
	∑	1.7	1.4	2
	σ	2.8	2.9	2.5
**AlignRT** Krengli et al. (2016) [26]	Mean	0.7	3.1	1.8
	SD	2.6	4.4	3.3
	∑	0.7	3.1	2.2
	σ	0.0	0.1	-0.4
**Virtual simulation** Nourzadeh et al. (2016) [76]	$\sum_{translation}$	2.6	2.4	2.6
	$\sigma_{translation}$	2	1.8	1.7
	$\sum_{rotation}$	1.1	0.6	0.5
	$\sigma_{rotation}$	1.1	0.6	0.5
**MRI** Kim et al. (2023) [53]	Mean± SD	1.06 ± 0.58	3.30 ± 1.85	2.32 ± 1.08
**Intrafractional errors (mm)**				
CBCT				
Sato et al. (2015) [41]	∑	0.3	0.6	0.7
	σ	0.7	1.1	1.3
	Mean ± SD	-0.1± 0.7	-0.1 ± 1.2	-0.3 ± 1.4
Hirose et al. (2018) [44]	$\sum_{soft\ tissue}$	0.25	0.66	0.68
	$\sigma_{soft\ tissue}$	0.50	1.21	1.08
Su et al. (2019) [32]	∑	0.3	1.0	0.5
	σ	0.8	1.1	1.6
Faccenda et al. (2023) [77]	Mean	−0.3 [−3.1–0.8]	0.0 [−4.2–3.7]	−0.7 [−3.5–1.9]

2D kV				
Groher et al. (2017) [78]	Σ	3.1	3.4	0.7
	σ	2.0	1.8	0.6
Hirose et al. (2018) [44]	$\sum_{FM}$	0.43	1.00	0.85
	σ_{FM}	0.75	1.66	1.47
Böckelmann et al. (2020) [38]	∑	0.44	0.69	0.80
	σ	1.91	2.30	2.27
	SD	1.95	2.43	2.36
MRI				
Willigenburg et al. (2022) [79]	Mean	0.09	-0.18	0.08
	$\Sigma_{translations}$	0.3	0.4	0.5
	$\sigma_{translations}$	0.8	1.3	1.4
	$\Sigma_{rotations}$	0.0	0.6	0.6
	$\sigma_{rotations}$	0.0	0.6	0.6
Kim et al. (2023) [53]	Mean± SD	2.12 ± 0.86	2.84 ± 0.88	2.24 ± 1.07
OTHER				
MVCT Iwama et al. (2014) [42]	Mean ± SD	0.03 ± 0.37	0.08 ± 0.13	0.52 ± 0.63
EM Mayyas et al. (2014) [25]	∑	0.6	1.5	1.3
	σ	1.4	2.4	2.6
US Faccenda et al. (2023) [77]	Mean	-0.2 [−1,5–0,8]	0,1 [−1,4] −1,5]	−0,3 [−1,7–1,4]

Note: In the studies presented in this table, a variety of errors have been reported beside the more common ones, such as: the group mean setup error (calculated by taking the mean of entire group setup error [20]); the residual error (defined as a discrepancy between the actual displacement from the planned position and the initial correction [25]) and the rigid body error (RBE) which was evaluated in terms of mean RBE and intrafraction and interfraction RBE. The intrafraction RBE variability was defined as the standard deviation of all RBE values during a single treatment fraction. Interfraction RBE variability was defined as the standard deviation of the mean daily RBE over the entire treatment course [73].

Abbreviations: CBCT = cone-beam computed tomography, 2D kV = two-dimensional kilovoltage planar imaging, EPID = Electronic Portal Imaging Device, US = ultrasounds, mm = millimeter, RBE = rigid body error, σ = random error and, Σ = systematic error, SD = standard deviation, FM = fiducial

marker, T + R = translation and rotational errors, LR = left right, SI = superior inferior, AP = anteroposterior.
