# Supplementary material for: From CBCT to MR-Linac in Image-Guided Prostate Cancer Radiotherapy Towards Treatment Personalization
Source: Curr Oncol. 2025 May 22;32(6):291. doi: 10.3390/curroncol32060291 (PMC12191943; doi:10.3390/curroncol32060291)
Supplement: Supplementary file 1 [file curroncol-32-00291-s001.zip › Table S2.pdf]

Table S2. PTV margin reduction in prostate cancer treatment according to the literature (studies are listed in chronological order)

Study (reference)	PTV margin planning (mm)	Method of PTV margin calculation	PTV margin based on IGRT errors (mm)		
			LR	SI	AP
Adamczyk et al. (2014) [64]	-	Van Herk formula	3.9	1.9	12.2
Iwama et al. (2014) [42]	5	Van Herk formula	0.6	1.1	0.78
Mayyas et al. (2014) [25]	-	Van Herk formula CBCT	7.8	8.3	9.7
		Van Herk formula kV	8.2	11.2	10.5
		Van Herk formula US	9.5	11.4	11.1
Oehler et al. (2014) [22]		Van Herk formula CBCT- low risk PCa	5.47	7.09	7.18/5.47
		Van Herk formula CBCT soft tissue matching- low risk PCa	5.88	7.16	7.69/6.09
		Van Herk formula CBCT bone matching- low risk PCa	5.95	9.00	9.45/8.20
		Van Herk formula CBCT balloon- low risk PCa	6.10	8.32	9.07/7.75
		Van Herk formula kV- low risk PCa	5.65	7.79	7.67/6.10
		Van Herk formula kV bone matching- low risk PCa	7.58	12.48	8.64/7.27
		Van Herk formula kV balloon- low risk PCa	7.58	12.48	8.64/7.27
		Van Herk formula CBCT - high risk PCa	7.39	9.67	10.52/6.39

		Van Herk formula CBCT soft tissue matching-high risk PCa	7.76	9.72	10.94/6.94
		Van Herk formula CBCT bone matching - high risk PCa	7.8	11.23	12.28/8.85
		Van Herk formula CBCT balloon matching - high risk PCa	7.95	10.71	11.99/8.44
		Van Herk formula kV - high risk PCa	7.53	10.19	10.88/6.94
		Van Herk formula kV bone matching - high risk PCa	7.88	11.24	12.18/8.68
		Van Herk formula kV balloon - high risk PCa	9.11	14.23	11.62/7.99
Shiraishi et al. (2014) [66]	-	Van Herk formula	2.1	4.3	4.4
		Yoda și Nakagawa formula	1.9	3.9	3.9
Chiesa et al. (2015) [67]	7/12 inferior		6	8	6
Sato et al. (2015) [41]	6/5 inferior și posterior	Van Herk formula bone matching	1.9	5.8	7.3
		Van Herk formula soft tissue matching	1.2	2.3	2.7
Rudat et al. (2016) [56]	8	Van Herk formula	4.1	8.1	6.6
		Van Herk nonIGRT	8.5	11.4	11.5
Drozdz et al. (2016) [51]	8/5 posterior	Van Herk formula NonIGRT Protocol 1	8.00	8.85	12.3
		Van Herk formula IGRT weekly Protocol 1	6.73	7.36	10.37
		Van Herk formula	4.45	5.07	6.94

		IGRT every 2nd day Protocol 1			
		Van Herk formula NonIGRT Protocol 2	5.97	6.79	8.50
		Van Herk formula IGRT weekly Protocol 2	4.90	5.70	7.25
		Van Herk formula IGRT every 2nd day Protocol 2	3.30	3.99	4.84
		Van Herk formula NonIGRT Protocol 3	5.56	5.06	8.54
		Van Herk formula IGRT weekly Protocol 3	4.79	4.40	7.31
		Van Herk formula IGRT every 2nd day Protocol 3	3.17	3.04	4.88
Jeong et al. (2016) [68]		Van Herk formula	8.2	6.45	5.25
Kanakavelu et al. (2016) [20]	CTV 10/8 GGL 5	Van Herk formula	5.02	4.56	4.52
Groher et al. (2017) [78]		Van Herk formula	8.5	9.0	1.9
Hirose et al. (2018) [44]	5/3 posterior	Van Herk formula 2D kV	1.6	3.7	3.2
		Van Herk formula CBCT soft tissue matching	2.2	4.8	4.1
		Van Herk formula CBCT	0.9	1.4	1.5
Van Nunen et al. (2018) [80]	-	online correction on bony anatomy prostate	6	9	11
		offline correction on bony anatomy	8	11	12

		prostate			
		online correction on the prostate fiducials prostate	5, with 8 to apex prostate and around SV		
		Isotropic margins prostate	10	10	10
	-	online correction on bony anatomy Lymph node	5	5	3
		offline correction on bony anatomy Lymph node	8	7	9
		online correction on the prostate fiducials Lymph node	6	10	12
		Isotropic margins Lymph node	10	10	10
Wang et al. (2018) [70]	-	Van Herk formula before correction	6.1	7.8	4.1
		Van Herk formula after correction	1.7	1.2	1.7
Ghaffari et al. (2019) [33]	-	Van Herk formula FM matching	4	3.3	3
		Van Herk formula NonIGRT	5.4	5.8	5.5
Ingrosso et al. (2019) [71]	6/5 posterior	Van Herk formula FM matching	2.5	5.6	3.9
Su et al. (2019) [32]		Van Herk formula bone matching	2.7 + 0.4	7.8 + 1.2	7.5 + 1.1
		Van Herk formula FM matching	1.5 + 0.2	3.5 + 0.5	2.7 + 0.4
		Van Herk formula tattoo matching	7.6 + 1.1	13.4 + 2.1	11.4 + 1.7
		Van Herk formula lymph node matching on bone	0.7 + 0.1	1.5 + 0.2	1.4 + 0.2

		Van Herk formula lymph node matching on FM	2.3 + 0.3	7.1 + 1.1	7.0 + 1.1
		Van Herk formula lymph node matching on tattoo	8.2 + 1.2	12.6 + 2.0	12.6+2.0
Böckelmann et al. (2020) [38]	15/10 posterior	Stroom formula adapted treatment plan	3.97	7.52	5.52
		Van Herk formula adapted treatment plan	4.57	8.71	6.37
		Stroom formula initial treatment plan	6.88	9.52	6.46
		Van Herk formula initial treatment plan	7.79	10.88	7.38
Kee Oh et al. (2020) [72]	-	Van Herk formula	5.96	4.01	6.43
Willigenburg et al. (2022) [79]		Van Herk formula MRI	1.2	2.4	2.6
Kim et al. (2023) [53]		Van Herk formula	2.21	5.40	5.16

Abbreviations: PCa = prostate cancer, CBCT = cone-beam computed tomography, 2D kV = two-dimensional kilovoltage planar imaging, mm = millimeter, FM = fiducial marker, LR = left right, SI = superior inferior, AP = anteroposterior, T = prostate follow-up time, Protocol *n* = using the first n fractions to correct the systematic error.
