# Supplementary material for: From CBCT to MR-Linac in Image-Guided Prostate Cancer Radiotherapy Towards Treatment Personalization
Source: Curr Oncol. 2025 May 22;32(6):291. doi: 10.3390/curroncol32060291 (PMC12191943; doi:10.3390/curroncol32060291)
Supplement: Supplementary file 1 [file curroncol-32-00291-s001.zip › Table S3.pdf]

**Table S3**. Dosimetric results based on the literature evaluated for PTV (studies are listed in chronological order)

| Study (reference) | PTV margin planning (mm) | Dosimetric parameter | Dosimetric values according to the IGRT technique | |
|---|---|---|---|---|
| | | | **Planned dose in CT simulation** | **Dose delivered based on CBCT/MRI/US scans** |
| Arnaund et al. (2014) [81] | 5 | D mean | 79.8 | 78.6 |
| | | D50% | 80.13 | 79.2 |
| | | D98% | 74.9 | 71.8 |
| | | D2% | 82.08 | 81.36 |
| | | D95% | 75.92 | 73.72 |
| | 10/5 posterior | D mean | 78.6 | 78.7 |
| | | D50% | 79.02 | 79.5 |
| | | D98% | 73 | 70 |
| | | D2% | 81.79 | 82.58 |
| | | D95% | 75.67 | 72.17 |
| Kasaova et al. (2014) [82] | 10 | D maximum | | 2D kV<br>82.7±2.2 |
| | | D minimum | | 69.4 ±3.3 |
| | | D maximum | | without 2D kV<br>82.2 ±2.1 |
| | | D minimum | | 58.2 ±15.8 |
| | 7 | D maximum | | 2D kV<br>82.2±2.3 |
| | | D minimum | | 68.0 ± 3.3 |
| | | D maximum | | without 2D kV<br>81.7± 2.4 |
| | | D minimum | | 52.0 ±14.4 |

| | | | | |
|---|---|---|---|---|
| Liu et al. (2014) [28] | 5/3posterior | V100Gy | 95.1 ± 0.1 | CBCT<br>85.2 ± 13.6 |
| | | V100Gy | 95.1 ± 0.1 | Adaptative radiotheray<br>92.9 ± 4.6 |
| | | V100Gy | 95.1 ± 0.1 | Reoptimization on daily anatomy<br>94.6 ± 2.4 |
| Onal et al (2014) [83] | 10/6 posterior | D2% | IMRT 6MV<br>82.51 | |
| | | D2% | VMAT 6MV<br>82.78 | |
| | | D2% | IMRT 10MV<br>82.27 | |
| | | D2% | VMAT 10MV<br>82.75 | |
| | | D2% | IMRT 15MV<br>82.19 | |
| | | D2% | VMAT 15MV<br>82.61 | |
| | | D95% | IMRT 6MV<br>78.13 | |
| | | D95% | VMAT 6MV<br>78.10 | |
| | | D95% | IMRT 10MV<br>78.12 | |
| | | D95% | VMAT 10MV<br>78.05 | |
| | | D95% | IMRT 15MV<br>78.11 | |

| | | | | |
|---|---|---|---|---|
| | | D95% | VMAT 15MV<br>78.11 | |
| | | D98% | IMRT 6MV<br>73.31 | |
| | | D98% | VMAT 6MV<br>77.29 | |
| | | D98% | IMRT 10MV<br>77.25 | |
| | | D98% | VMAT 10MV<br>77.20 | |
| | | D98% | IMRT 15MV<br>77.20 | |
| | | D98% | VMAT 15MV<br>77.25 | |
| Chiesa et al. (2015) [67] | 7/12 inferior | | Dosimetric impact on V95% coverage, comparing planning and re-planning phases. | |
| | | D mean | -0.2 | -0.2 |
| | | D maximum | 0.4 | 0.1 |
| | | D minimum | −1.6 | −1.1 |
| | 7 | D mean | 0.2 | -1.1 |
| | | D maximum | 0.2 | 0.1 |
| | | D minimum | -3.5 | −8.9 |
| Ariyaratne et al. (2016) [50] | 7 | V95Gy daily | | 94.36 |
| | | V95Gy weekly | | 90.97 |
| Li et al. (2016) [36] | 7/5 posterior | D95% | Planned dose with IMRT CT | Delivery dose with IMRT CBCT |
| | | | 77.41± 1.06 | 76.63 ± 6.95 |
| | 5/4 posterior | D95% | VMAT CT | VMAT CBCT |
| | | | 69.84± 0.21 | 69.24 ± 0.57 |

| | | | | |
|---|---|---|---|---|
| Moteabbed et al (2016) [58] | | D98% Proton therapy | 98.6 ± 2.1 | 95.9 ± 5.0 |
| | | D98% IMRT | 98.1 ± 0.6 | 94.2 ± 3.3 |
| Scobiola et al. (2016) [86] | 5/3 posterior | D98% | Tomotherapy 95,3 ± 1,9 | |
| | | D2% | 103,3 ± 0,5 | |
| | | D98% | IMRT 95,5 ± 0,6 | |
| | | D2% | 102,5 ± 0,4 | |
| | | D98% | VMAT 95,3 ± 0,6 | |
| | | D2% | 102,4 ± 0,3 | |
| | | D98% | Proton therapy 96,7 ± 0,4 | |
| | | D2% | 102,5 ± 0,4 | |
| Park et al. (2017) [84] | 3 | D2% | | 89.1 ± 0.6 Gy |
| | | D95% | | 81.3 ± 0.1 Gy |
| | | D98% | | 80.4 ± 0.4 Gy |
| | | D mean | | 85.4 ± 0.5 Gy |
| | 7 | D2% | | 84.5 ± 0.3 |
| | | D95% | | 82.5 ± 0.3 |
| | | D98% | | 82.0 ± 0.3 |
| | | D mean | | 83.5 ± 0.3 |
| Tøndel et al. (2017) [85] | 15 | D mean | | 74.5 (74.1–74.8) Gy |
| | 7 | D mean | | 76.2 (76.1–76.4) Gy |
| Van Nunen et al. (2018) [80] | 6/9/11 | D mean | | 67.1 Gy |
| | 8/11/12 | D mean | | 67.2 Gy |

| | | | | |
|---|---|---|---|---|
| | 5, with 8 to apex prostate and around SV | D mean | | 67.3 Gy |
| | 10 | D mean | | 67.2 Gy |
| | 5/5/7 | D mean | | 50.6 Gy |
| | 8/7/9 | D mean | | 51 Gy |
| | 6/10/12 | D mean | | 50.8 Gy |
| | 10 | D mean | | 51.1 Gy |
| Rossi et al. (2018) [87] | 3 | V100% | 95.3 | |
| | | D98% | 35.8 | |
| | 5 | V100% | 92.7 | |
| | | D98% | 33.7 | |
| Gozal et al. (2020) [39] | | D2% | 3D CRT 103.5 | |
| | | D95% | 3D CRT 95.74 | |
| | | D2% | IMRT 105.87 | |
| | | D95% | IMRT 95.28 | |
| | | D2% | VMAT 106.28 | |
| | | D95% | VMAT 95.15 | |
| | | D2% | Tomotherapy 101.4 | |
| | | D95% | Tomotherapy 94.47 | |
| Mannerberg et al. (2020) [16] | 7 | | Difference between hypofractionated and ultra-hypofractionated plane | |

| | | | | |
|---|---|---|---|---|
| | | D95% | 2.8 (−36.4 – 0.06) | |
| | | D98% | −5.7 (−52.7 – −0.11) | |
| | 5 | D95% | −2.9 (−39.2 – 0.09) | |
| | | D98% | −5.2 (−51.5 – −0.26) | |
| | 3 | D95% | -3.1 (−42.4 – 0.07) | |
| | | D98% | -5.0 (−53.6 – −0.06) | |
| Kinhikar et al. (2021) [5] | 10SI/7 LR and AP | D maximum | VMAT 67.34±1.74Gy | |
| | | D maximum | Tomotherapy 68.67±3.1Gy | |
| | | D maximum | IMRT 66.24±3.16Gy | |
| | | D maximum | 3D CRT 62.09±2.44Gy | |
| | | D98% | VMAT 59.54±1.35 | |
| | | D98% | Tomotherapy 58.27±3.85 | |
| | | D98% | IMRT 57.83±2.29 | |
| | | D98% | 3D CRT 57.83±2.29 | |
| Da Silva et al. (2021) [1] | 6/5 posterior | | Planned dose with VMAT | Planned dose with IMRT |
| | | V 40Gy | 100 ± 0.0 | 103 ± 8 |
| | | V 25Gy | 100 ± 0.0 | 114 ± 9 |
| | 4/3 superior, inferior and posterior | V 40Gy | | 81 ± 9 |
| | | V 25Gy | | 89 ± 11 |

| | | | | |
|---|---|---|---|---|
| Wang et al. (2021) [88] | 5 | D90% daily | 104.8 | 103.32 |
| | | D95% daily | 102.65 | 100.99 |
| | | D90% weekly | 104.8 | 103.52 |
| | | D95% weekly | 102.65 | 101.06 |
| Pokhler et al. (2021) [40] | 3 | D95% | Halcyon VMAT<br>36.25 ± 0 | |
| | | D95% | Truebeam VMAT<br>36.25 ± 0 | |
| Tetar et al. (2022) [30] | 3 | D95% | CTV prostate-RECALC<br>96.1% | |
| | 5 | D95% | CTV prostate-RECALC<br>99.2% | |
| | 3 | D95% | CTV prostate-REOPT<br>99.5% | |
| | 3 | D95% | CTV Seminal vescles V-RECALC<br>83.6% | |
| | 5 | D95% | CTV Seminal vescles -RECALC<br>98.9% | |
| | 3 | D95% | CTV Seminal vescles -REOPT<br>99.5% | |
| Bartlett et al. (2023) [89] | 5 | V100% | IMRT<br>79.99 | |
| | | V100% | VMAT<br>80.91 | |
| | | V100% | VMAT partial arc<br>80.21 | |

| | | | | |
|---|---|---|---|---|
| Faccenda et al. (2023) [77] | 2 | Differences between planned and administered dose | | |
| | | D2% | −0.3% (−1.1–0.2 | |
| | | D95% | -2.7% (−11.9–−0.2) | |
| Fathy et al. (2023) [90] | 7/4 posterior | D2% | VMAT FF 63.8 | |
| | | D95% | VMAT FF 59.3 | |
| | | D medie | VMAT FF 61.5 Gy | |
| | | D2% | VMAT FFF 64 | |
| | | D95% | VMAT FFF 58.9 | |
| | | D mean | VMAT FFF 61.5 Gy | |
| | | D2% | VMAT MLCi2 63.3 | |
| | | D95% | VMAT MLCi2 58.2 | |
| | | D mean | VMAT MLCi2 60.9 Gy | |
| Gao et al. (2023) [91] | 3 | V95% | 97.83 ± 0.13% | |
| | 3 | V95% | MRI position verification 99.93 ± 0.30%, | |
| | 3 | V95% | Post-MRI 98.69 ± 1.85% | |
| Onal et al. (2024) [64] | 6/5 posterior | D98% | 36.13 ± 0.27 | |
| | 3 | D98% | 35.90 ± 0.22 | |
| | 6/5 posterior | D mean | 37.11 ± 0.20 | |

| | | | | |
|---|---|---|---|---|
| | 3 | D mean | 37.07 ± 0.19 | |
| Byrne et al. (2024) [92] | 10 | D95% prostate | IGRT<br>60.2Gy | |
| | | | Adaptivewithverification image<br>61.3Gy | |
| | | | Adaptive without verification image<br>61.0Gy | |
| | | D95% seminal vesicles | IGRT<br>60.0Gy | |
| | | | Adaptive with verification image<br>61.0Gy | |
| | | | Adaptive without verification image<br>60.8Gy | |

**Abbreviations:** IMRT = intensity modulated radiation therapy; VMAT = volumetric modulated arc therapy; 3D CRT = three-dimensional conformal radiation therapy; mm = millimeters; MLCi = multileaf collimator; FF = flattening filter; FFF = flattening filter free; MV = megavoltage, Gy = Gray, CBCT = cone-beam computed tomography, CT = computed tomography, US = ultrasound, MRI = magnetic resonance imaging
