# Supplementary material for: From CBCT to MR-Linac in Image-Guided Prostate Cancer Radiotherapy Towards Treatment Personalization
Source: Curr Oncol. 2025 May 22;32(6):291. doi: 10.3390/curroncol32060291 (PMC12191943; doi:10.3390/curroncol32060291)
Supplement: Supplementary file 1 [file curroncol-32-00291-s001.zip › Table S4.pdf]

Table S4. Dosimetric parameters evaluated for the OARs during prostate cancer radiotherapy (studies are listed in chronological order)

**Study (reference)**	**PTV margin planning (mm)**	**Dosimetric parameter of rectum**	**Dosimetric values**	
			**Planned dose in CT simulation**	**Dose delivered based on CBCT/MRI/US scans**
**Rectum**				
Arnaud et al. (2014) [81]	5	D mean	50.8	49
		D98%	8.61	8.2
		V70%	9.8	11.7
		V75%	1.8	1.22
	10/ 5 posterior	D mean	55.3	54.8
		D98%	10.91	16.35
		V70%	9.85	9.64
		V75%	2.9	2.92
Elith et al. (2014) [93]	10/6 posterior	V5%	VMAT partial arc 89.4	
		V40%	VMAT partial arc 41.1	
		V60%	VMAT partial arc 21.4	
		V75%	VMAT partial arc 3.8	
		V5%	VMAT 1 arc 89.9	

		V40%	VMAT 1 arc<br>46.4	
		V60%	VMAT 1 arc<br>21.3	
		V75%	VMAT 1 arc<br>1.9	
		V5%	VMAT 1 arc+ partial arc<br>89.9	
		V40%	VMAT 1 arc+ partial arc<br>41.0	
		V60%	VMAT 1 arc+ partial arc<br>20.4	
		V75%	VMAT 1 arc+ partial arc<br>1.9	
		V5%	VMAT 2 arcs<br>90.0	
		V40%	VMAT 2 arcs<br>44.0	
		V60%	VMAT 2 arcs<br>20.7	
		V75%	VMAT 2 arcs<br>1.9	
Hirose et al. (2014) [65]	8/5 posterior	V100%	0	Tattoo<br>1.1
		V90%	11.5	Tattoo<br>14.5
		V100%	0	CBCT

				0.8
		V90%	11.5	CBCT<br>14.8
	5	V100%	0	0.9
		V90%	11.6	14
		V100%	0	0.6
		V90%	11.6	14.7
Kasaova et al. (2014) [82]	10	D maximum		2D kV<br>76.6 ± 0.7
		D mean		47.5 ± 6.8
		D maximum		Without 2D kV<br>76.9 ± 1.9
		D mean		47.5 ± 6.4 4
	7	D maximum		2D kV<br>77.2 ± 1.4
		D mean		41.9 ± 12.8
		D maximum		Without 2D kV<br>76.4 ± 3.2
		D mean		41.7 ± 13.4
Liu et al. (2014) [28]	5/3 posterior	V45Gy	44.9 ± 8.1	CBCT<br>56.5 ± 17.7
		V45Gy	44.9 ± 8.1	Adaptative radiotherapy<br>48.2 ± 11.8
		V45Gy	44.9 ± 8.1	Reoptimization on daily anatomy<br>42.5 ± 9.6
		V60Gy	16.8 ± 7.8	CBCT<br>32.8 ± 18.5

		V60Gy	16.8 ± 7.8	Adaptative radiotherapy<br>22.6 ± 9.7
		V60Gy	16.8 ± 7.8	Reoptimization on daily anatomy<br>16.5 ± 7.4
Onal et al. (2014) [83]	10/6 posterior	V40%	IMRT 6MV<br>53.10	
		V60%	IMRT 6MV<br>25.44	
		D 2cc	IMRT 6MV<br>78.10	
		D mean	IMRT 6MV<br>42.89	
		V40%	VMAT 6MV<br>49.20	
		V60%	VMAT 6MV<br>23.36	
		D 2cc	VMAT 6MV<br>77.86 Gy	
		D mean	VMAT 6MV<br>41.79 Gy	
		V40%	IMRT 10MV<br>54.44	
		V60%	IMRT 10MV<br>25.27	
		D 2cc	IMRT 10MV<br>78.17 Gy	
		D mean	IMRT 10MV	

			43.25 Gy	
		V40%	VMAT 10MV<br>50.66	
		V60%	VMAT 10MV<br>23.82	
		D 2cc	VMAT 10MV<br>77.38 Gy	
		D mean	VMAT 10MV<br>41.85 Gy	
		V40%	IMRT 15MV<br>55.64	
		V60%	IMRT 15MV<br>25.63	
		D 2cc	IMRT 15MV<br>78.11 Gy	
		D mean	IMRT 15MV<br>43.72 Gy	
		V40%	VMAT 15 MV<br>52.73	
		V60%	VMAT 15 MV<br>24.76	
		D 2cc	VMAT 15 MV<br>77.45 Gy	
		D mean	VMAT 15 MV<br>42.70 Gy	
Cakir et al. (2015) [94]	5	V20%	3D CRT<br>41.3	

		V20%	IMRT<br>40.9	
		V20%	VMAT<br>48.8	
		V50%	3D CRT<br>16.9	
		V50%	IMRT<br>14.4	
		V50%	VMAT<br>15.9	
		V70%	3D CRT<br>6.3	
		V70%	IMRT<br>5.9	
		V70%	VMAT<br>5.6	
Oates et al. (2015) [95]	10/6 posterior	V50 Gy	9±5	
	6	V50 Gy adapted	4±27	
Ariyaratne et al. (2016) [50]	7mm	V 50Gy daily		32.48
		V65% daily		18.01
		D man daily		36.56Gy
		D2cc daily		73.37Gy
		V 50Gy weekly		34.96
		V65% weekly		20.45
		D mean weekly		37.68Gy
		D2cc weekly		73.35Gy
	3	V 50Gy daily		21.14
		V65% daily		9.65
		D mean daily		28.99Gy

		D2cc daily		70.43Gy
	5	V 50Gy daily		26.34
		V65% daily		13.62
		D mean daily		32.20Gy
		D2cc daily		72.39Gy
Li et al. (2016) [36]	7/5 posterior	R D25	60.39 ± 3.49	61.78 ± 7.14
		R D35	51.49 ± 6.53	52.27 ± 7.11
		R D50	39.20 ± 1.71	41.11 ± 0.56
	5/4 posterior	R D25	50.46 ± 8.88	50.13 ± 10.91
		R D35	42.22 ± 7.90	42.26 ± 8.66
		R D50	34.23 ± 5.51	32.61 ± 4.73
Moteabbed et al. (2016) [58]	5/4 posterior	V75%	Proton therapy 4.7 ± 1.2	Proton therapy 4.0 ± 1.3
		V70%	7.5 ± 1.6	6.6 ± 1.8
		V50%	16.4 ± 3.7	15.9 ± 4.8
		V40%	21.0 ± 5.3	21.0 ± 7.0
		V75%	IMRT 3.5 ± 1.2	IMRT 3.8 ± 2.3
		V70%	5.5 ± 1.6	5.9 ± 2.9
		V50%	13.7 ± 3.7	14.4 ± 4.8
		V40%	19.9 ± 5.8	20.8 ± 6.4
Scobioala et al. (2016) [86]	5/3 posterior	V10%	Tomotherapy 59.1 ± 17.9	
		V30%	24.8 ± 7.6	
		V50%	12.8 ± 4.0	
		V70%	1.1 ± 0.7	
		D mean	17.3 ± 3.9 Gy	
		D maximum	78.3 ± 1.8 Gy	

		D1 cc	67.5 ± 4.1 Gy	
		V10%	IMRT<br>62.7 ± 16.6	
		V30%	32.8 ± 5.7	
		V50%	17.3 ± 2.5	
		V70%	1.5 ± 0.8	
		D mean	20.3 ± 3.4 Gy	
		D maximum	75.9 ± 1.2 Gy	
		D1 cc	68.9 ± 2.4 Gy	
		V10%	VMAT<br>69.0 ± 18.7	
		V30%	41.7 ± 11.3	
		V50%	23.5 ± 7.5	
		V70%	3.4 ± 1.8	
		D mean	24.5 ± 5.3 Gy	
		D maximum	77.3 ± 1.5 Gy	
		D1 cc	71.5 ± 2.5Gy	
		V10%	Proton therapy<br>24.6 ± 9.2	
		V30%	15.7 ± 5.8	
		V50%	9.3 ± 3.8	
		V70%	1.0 ± 0.8	
		D mean	9.0 ± 3.4Gy	
		D maximum	76.4 ± 2.2Gy	
		D1 cc	65.9 ± 4.2Gy	
Park et al. (2017) [84]	3	V20%		54.3 ± 4.4
		V50%		9.0 ± 3.0
		R V65%		9.0 ± 3.0

		V 70%		5.2 ± 1.8
		V 75%		2.2 ± 0.9
		V80%		0.3 ± 0.4
	7	V20%		68.9 ± 3.4
		V50%		52.5 ± 2.4
		V65%		25.6 ± 5.4
		V 70%		19.1 ± 4.0
		V 75%		13.4 ± 3.4
		V80%		8.2 ± 2.8
Nakamura et al. (2017) [2]	9/6 posterior 2D kV	V75Gy		0.0
		V70Gy		1.5
		V60Gy		13.0
		V50Gy		21.3
	6/5posterior/7.5superior si inferior CBCT	V75Gy		0.1
		V70Gy		2.5
		V60Gy		9.8
		V50Gy		16.4
Tøndel et al. (2017) [85]	15	V70%		18.5 (16.3–20.62)
		V60%		36.2 (32.7–39.7)
		V50%		44.9 (40.8–49.0)
	7	V70%		11.5 (10.3–12.7)
		V60%		22.6 (20.4–24.8)
		V50%		29.8 (26.9–32.6)
Van Nunen et al. (2018) [80]	6/9/11	D mean		37.5
	8/11/12	D mean		37.9
	5, with 8 to apex prostate and around SV	D mean		31.9
	10	D mean		37.7

Rossi et al. (2018) [87]	3	D1cc	29.4Gy	
		V 60%	1.5	
		V40%	4.9	
		D mean	6.6Gy	
	5	D1cc	32.2Gy	
		V 60%	3.3	
		V40%	9.3	
		D mean	9.2Gy	
Zhao et al. (2019) [4]	5	V65Gy		14.35
		V40Gy		39.50
	3	V65Gy		8.90
		V40Gy		28.54
Gozal et al (2020) [39]		V75 Gy	3D CRT 61.22±18.80	
		V75 Gy	IMRT 13.56±1.21	
		V75 Gy	VMAT 11.64±3.17	
		V75 Gy	Tomotherapy 13.53±0.84	
Mannerberg et al. 2020 [16]			Difference between hypofractionated and ultra-hypofractionated plan	
	7	D15%	−3.6 (−52.3 – 28.7)	
	5	D15%	−3.6 (−56.1 – 41.7)	
	3	D15%	−2.6 (−56.0 – 43.0)	
Kinhikar et al. (2021) [5]	10SI/7 LR and AP	V40 %	VMAT 55.08±24.99	
		V40 %	Tomotherapy 41.56±16.35	

		V40 %	IMRT<br>29.65±17.03	
		V40 %	3D CRT<br>60.42±26.45	
		D maximum	VMAT<br>65.63±2.01Gy	
		D maximum	Tomotherapy<br>67.01±2.51Gy	
		D maximum	IMRT<br>64.31±3.11Gy	
		D maximum	3D CRT<br>62.06±2.43Gy	
Pokhler et al. (2021) [40]	3	D1cc	Halcyon VMAT<br>32.25 ± 4.77	
		D1cc	Truebeam VMAT<br>32.46 ± 4.55	
		D50%	Halcyon VMAT<br>12.77 ± 4.26	
		D50%	Truebeam VMAT<br>13.39 ± 4.88	
Tetar et al. (2022) [30]	3	V36.25Gy	CTV - RECALC<br>0.12 cc	
	5	V36.25Gy	CTV - RECALC<br>0.29 cc	
	3	V36.25Gy	CTV -REOPT<br>0.39 cc	

Yagihashi et al. (2022) [96]	8/5 posterior	V30Gy	IMRT robust<br>48.8 ± 12.4	
		V57Gy	27.6 ± 8.6	
		V30Gy	IMRT<br>49.8 ± 12.7	
		V57Gy	29.9 ± 9.3	
	10/7 posterior	V30Gy	IMRT robust<br>52.0 ± 13.2	
		V57Gy	32.2 ± 9.5	
		V30Gy	IMRT<br>53.1 ± 13.2	
		V57Gy	34.7 ± 10.3	
	5/3 posterior	V30Gy	IMRT robust<br>43.5 ± 10.9	
		V57Gy	20.3 ± 7.0	
		V30Gy	IMRT<br>44.3 ± 11.5	
		V57Gy	22.5 ± 7.7	
Bartlett et al. (2023) [89]	5	D mean	IMRT<br>40.91Gy	
		D1cc	74.36Gy	
		V 75%	7.7	
		V70%	11.8	
		V 65%	15.6	
		V50%	29.1	
		V 40%	47.5	
		D mean	VMAT<br>45.24Gy	
		D1cc	76.39Gy	

		V 75%	8.4	
		V70%	12.9	
		V 65%	17.3	
		V50%	37.1	
		V 40%	66.1	
		D mean	VMAT partial arc<br>41.85Gy	
		D1cc	75.18Gy	
		V 75%	8.1	
		V70%	12.0	
		V 65%	15.7	
		V50%	29.8	
		V 40%	50.0	
Faccenda et al. (2023) [77]	3	V5%	−4.7% (−27.7–12.0)	
		V10%	−5.1% (−33.5–14.6)	
		V20%	−3.7% (−31.3–15.5)	
		V50%	−1.5% (−18.9–16.1)	
Fathy et al. (2023) [90]	7/4 posterior	V 60%	VMAT FF<br>4.2	
		D mean	VMAT FF<br>37.1Gy	
		V 60%	VMAT FFF<br>3.95	
		D mean	VMAT FFF<br>36.6Gy	
		V 60%	VMAT MLCi2<br>2.96	

		D mean	VMAT MLCi2<br>37.2 Gy	
Gao et al. (2023) [91]	3	V38Gy	MRI position verification<br>0.23 ± 0.28 cc	
	3	V38Gy	Post MRI<br>0.39 ± 0.52 cc	
Polizzi et al. (2024) [97]	7/4 posterior	V95%	CT planning<br>4.90 cc	
			CBCT prostate alignment<br>6.58 cc	
			CBCT rectum alignment<br>5.7 1cc	
**Bladder**				
Arnaud et al. (2014) [81]	5	D mean	40.3	40.5
		D98%	15	6.66
		V70%	23.04	17
		V75%	16.7	10
	10/5 posterior	D mean	44.2	44.5
		D98%	3.1	4.3
		V70%	26.4	25.1
		V75%	10.33	5.19
Elith et al. (2014) [93]	10/6 posterior	V5%	VMAT partial arc<br>66.4	
		V40%	VMAT partial arc	

			28.3	
		V60%	VMAT partial arc<br>17.4	
		V75%	VMAT partial arc<br>5.9	
		V5%	VMAT 1 arc<br>68.4	
		V40%	VMAT 1 arc<br>29.3	
		V60%	VMAT 1 arc<br>17.0	
		V75%	VMAT 1 arc<br>4.3	
		V5%	VMAT 1arc+ partial arc<br>68.0	
		V40%	VMAT 1arc+ partial arc 27.9	
		V60%	VMAT 1arc+ partial arc 17.0	
		V75%	VMAT 1arc+ partial arc 5.2	
		V5%	VMAT 2 arcs<br>68.7	
		V40%	VMAT 2 arcs<br>28.6	
		V60%	VMAT 2 arcs<br>17.3	

		V75%	VMAT 2 arcs<br>4.6	
Hirose et al. (2014) [65]	8/5 posterior	V100%	8.3	Tattoo<br>8.5
		V90%	19.5	Tattoo<br>20.8
		V100%	8.3	CBCT<br>8.8
		V90%	19.5	CBCT<br>21.2
	5	V100%	5.4	Tattoo<br>5.9
		V90%	16	Tattoo<br>16.5
		V100%	5.4	CBCT<br>5.9
		V90%	16	CBCT<br>17
Kasaova et al. (2014) [82]	10	D maximum		2D kV<br>77.5 ± 1.4
		D mean		39.5 ± 16.0
		D maximum		Without 2D kV<br>77.1 ±4.1
		D mean		39.2 ± 19.5
	7	D maximum		2D kV<br>65.4 ± 6.1
		D mean		30.2 ± 20.7
		D maximum		Without 2D kV<br>77.1 ±4.4

		D mean		30.3 ± 22.6
Onal et al. (2014) [83]	10/6 posterior	V40%	IMRT 6MV 69.85	
		V60%	IMRT 6MV 40.01	
		D mean	IMRT 6MV 49.75 Gy	
		V40%	VMAT 6MV 63.35	
		V60%	VMAT 6MV 36.61	
		D mean	VMAT 6MV 47.10 Gy	
		V40%	IMRT 10MV 69.39	
		V60%	IMRT 10MV 38.88	
		D mean	IMRT 10MV 49.46 Gy	
		V40%	VMAT 10MV 62.43	
		V60%	VMAT 10MV 36.31	
		D mean	VMAT 10MV 47.08 Gy	
		V40%	IMRT 15MV 69.26	
		V60%	IMRT 15MV 38.99	

		D mean	IMRT 15MV<br>49.67 Gy	
		V40%	VMAT 15 MV<br>61.92	
		V60%	VMAT 15 MV<br>36.51	
		D mean	VMAT 15 MV<br>47.10 Gy	
Cakir et al. (2015) [94]	5	V20%	3D CRT<br>24.2	
		V20%	IMRT<br>49.4	
		V20%	VMAT<br>48.8	
		V50%	3D CRT<br>11.8	
		V50%	IMRT<br>20.3	
		V50%	VMAT<br>18.7	
		V70%	3D CRT<br>7.0	
		V70%	IMRT<br>8.9	
		V70%	VMAT<br>8.1	
	10/6 posterior	V50 Gy	32±5	

Oates et al. (2015) [95]	6	V50 Gy adapted	43±19	
Ariyaratne et al. (2016) [50]	7	V65Gy daily		10.32
		D mean daily		23.13Gy
		V65Gy weekly		9.9
		D mean weekly		22.82Gy
	3	V65Gy daily		6.18
		D mean daily		18.45Gy
	5	V65Gy daily		8.34
		D mean daily		20.75Gy
Li et al. (2016) [36]	7/5 posterior	D25 Gy	54.07 ± 20.11	52.80 ± 20.28
		D35 Gy	43.56 ± 21.70	41.97 ± 20.96
		D50 Gy	32.70 ± 18.90	31.48 ± 17.95
	5/4 posterior	D25 Gy	47.70 ± 4.57	41.95 ± 13.75
		D35 Gy	38.36 ± 3.30	34.28 ± 13.63
		D50 Gy	27.03 ± 4.69	25.07 ± 12.30
Moteabbed et al. (2016) [58]	5/4 posterior	V75%	Proton therapy 6.9 ± 3.1	Proton therapy 6.0 ± 4.2
		V70%	9.2 ± 4.4	8.7 ± 5.7
		V50%	15.2 ± 6.9	16.0 ± 10.2
		V45%	18.4 ± 8.2	21.0 ± 13.2
		V75%	IMRT 3.7 ± 2.4	IMRT 3.9 ± 2.9
		V70%	5.6 ± 3.6	6.1 ± 3.8
		V50%	11.5 ± 6.6	13.1 ± 7.2
		V45%	17.3 ± 9.5	19.4 ± 10.3
	5/3 posterior	V10%	Tomotherapy	

Scobiola et al. (2016) [86]			42.0 ± 23.8	
		V30%	26.2 ± 18.6	
		V50%	16.8 ± 12.5	
		V70%	6.0 ± 4.9	
		D mean	17.4 ± 10.1 Gy	
		D maximum	82.5 ± 2.8 Gy	
		D1 c^3	76.3 ± 8.2 Gy	
		V10%	IMRT 39.3 ± 23.3	
		V30%	23.5 ± 16.4	
		V50%	15.6 ± 11.6	
		V70%	5.4 ± 4.8	
		D mean	15.8 ± 9.6 Gy	
		D maximum	80.0 ± 2.8 Gy	
		D1 cc	76.2 ± 6.3 Gy	
		V10%	VMAT 46.8 ± 23.2	
		V30%	27.0 ± 19.1	
		V50%	17.0 ± 13.3	
		V70%	6.4 ± 4.8	
		D mean	17.9 ± 10.1 Gy	
		D maximum	80.9 ± 3.1 Gy	
		D1 cc	76.3 ± 8.1 Gy	
		V10%	Proton therapy 30.2 ± 18.5	
		V30%	21.2 ± 14.7	
		V50%	15.3 ± 11.5	

		V70%	5.3 ± 4.7	
		D mean	13.3 ± 9.1Gy	
		D maximum	80.9 ± 1.7 Gy	
		D1 cc	76.2 ± 6.0 Gy	
Park et al. (2017) [84]	3	V30%		29.9 ± 12.4
		V 55%		15.9 ± 10.1
		V65%		6.0 ± 4.2
		V70%		4.0 ± 3.1
		V80%		0.9 ± 1.2
	7	V30%		48.0 ± 13.9
		V 55%		29.5 ± 17.0
		V65%		13.9 ± 8.2
		V70%		10.9 ± 6.7
		V80%		5.5 ± 3.7
Nakamura et al. (2017) [2]	9/6 posterior 2D kV	V75Gy		0.6
		V70Gy		9.4
		V60Gy		17.2
		V50Gy		23
	6/5posterior /7.5superior and inferior	V75Gy		5.3
		V70Gy		11.8
		V60Gy		18.5
		V50Gy		24.3
Tøndel et al. (2017) [85]	15	V70%		46.3 (42.5–50.0)
		V60%		74.0 (69.5–78.6)
		V50%		83.6 (78.6–88.5)
	7	V70%		30.4 (27.5–33.3)
		V60%		45.1 (41.6–48.5)
		V50%		53.8 (50.0–57.7)
	6/9/11	D mean		44.8

Van Nunen et al. (2018) [80]	8/11/12	D mean		46.3
	5, with 8 to apex prostate and around SV	D mean		44.3
	10	D mean		46.2
Rossi et al. (2018) [87]	3	$D1c^3$	37.2Gy	
		D mean	8.4Gy	
	5	$D1c^3$	37.6Gy	
		D mean	9.3Gy	
Gozal et al. (2020) [39]		V75Gy	50.17±29.32	
		V75Gy	20.19±4.16	
		V75Gy	19.36±0.94	
		V75Gy	19.93±4.15	
Mannerberg et al. (2020) [16]			Difference between hypofractionated and ultra-hypofractionated plan	
	7	D_{mean}	−12.6 (−32.2 – 13.5)	
	5	D_{mean}	−11.8 (−32.1 – 17.1)	
	3	D_{mean}	−10.2 (−32.7 – 26.7)	
Kinhikar et al. (2021) [5]	10 SI /7 LR and AP	V40%	VMAT 26.58±20.18	
		V40%	Tomotherapy 28.22±22.49	
		V40%	IMRT 21.02±15.38	
		V40%	3D CRT 28.70±20.04	
		D maximum	VMAT 66.64±1.94Gy	
		D maximum	Tomotherapy 67.7±2.64Gy	

		D maximum	IMRT<br>65.42±3.11Gy	
		D maximum	3D CRT<br>60.62±2.38Gy	
Pokhler et al. (2021) [40]	3	D1cc	Halcyon VMAT<br>37.18 ± 0.24	
		D1cc	Truebeam VMAT<br>37.28 ± 0.29	
		D50%	Halcyon VMAT<br>3.50 ± 2.64	
		D50%	Truebeam VMAT<br>3.29 ± 2.39	
Tetar et al. (2022) [30]	3	V36.25Gy	CTV - RECALC 0.19 cc	
	5	V36.25Gy	CTV - RECALC 0.37 cc	
	3	V36.25Gy	CTV -REOPT 0.14 cc	
Yagihashi et al. (2022) [96]	5/3 posterior	40Gy	IMRT robust<br>31.1 ± 10.3	
		50Gy	22.2 ± 7.7	
		60Gy	9.2 ± 3.6	
		40Gy	IMRT<br>32.7 ± 10.8	
		50Gy	23.8 ± 8.1	
		60Gy	10.5 ± 3.7	

	8/5 posterior	40Gy	IMRT robust<br>36.0 ± 10.9	
		50Gy	26.4 ± 8.2	
		60Gy	12.0 ± 4.3	
		40Gy	IMRT<br>37.7 ± 11.1	
		50Gy	28.4 ± 8.7	
		60Gy	13.3 ± 4.6	
	10/7 posterior	40Gy	IMRT robust<br>39.4 ± 11.2	
		50Gy	29.3 ± 8.6	
		60Gy	14.0 ± 4.8	
		40Gy	IMRT<br>41.3 ± 11.7	
		50Gy	31.5 ± 9.0	
		60Gy	15.4 ± 5.3	
Bartlett et al. (2023) [88]	5	D mean	IMRT<br>36.12Gy	
		D1c^3	78.66Gy	
		V 75%	7.2	
		V70%	9.5	
		V 65%	11.9	
		V50%	21.0	
		V 40%	37.4	
		D mean	VMAT<br>39.22Gy	
		D1c^3	78.71Gy	
		V 75%	6.7	
		V70%	9.0	

		V 65%	11.3	
		V50%	23.1	
		V 40%	45.4	
		D mean	VMAT partial arc<br>35.51Gy	
		$D1c^{3}$	77.86Gy	
		V 75%	6.1	
		V70%	8.2	
		V 65%	10.4	
		V50%	20.1	
		V 40%	36.5	
Faccenda et al. (2023) [77]	2	V 0.035 cc	−0.8% (−2.4–0.4)	
		B V10%	−4.6% (−27.6–20.5)	
		B V40%	−0.9% (−52.7–234.4)	
Fathy et al. (2023) [90]	7/4 posterior	V60%	VMAT FF<br>9.6	
		D mean	VMAT FF<br>36.7 Gy	
		V60%	VMAT FFF<br>9	
		D mean	VMAT FFF<br>37.4 Gy	
		V60%	VMAT MLCi2<br>7.2	
		D mean	VMAT MLCi2<br>37.7 Gy	

Gao et al. (2023) [91]	3	V37Gy		MRI position verification<br>2.44 ± 1.15	
	3	V37Gy		Post MRI<br>3.27 ± 1.85 cc,	
Onal et al. (2024) [64]	6/5 posterior	V36.25 Gy		4.76 ± 2.77	
	3	V36.25 Gy		2.43 ± 1.87 Gy	
**Femoral heads**					
Elith et al. (2014) [94]	10/6 posterior	Right	VMAT partial arc<br>V20%	56.2 (48.6–63.8)	
			VMAT partial arc<br>V40%	2.3 (0.6–4.0)	
			VMAT 1 arc<br>V20%	41.2 (29.9–52.5)	
			VMAT 1 arc<br>V40%	0.4 (-0.05 - 0.9)	
			VMAT 1arc+ partial arc<br>V20%	39.0 (30.8–47.3)	
			VMAT 1arc+ partial arc<br>V40%	0.4 (-0.04 - 0.8)	
			VMAT 2 arc<br>V20%	34.4 (26.0–42.8)	

			VMAT 2 arc V40%	0.4 (-0.08 - 0.8)	
		Left	VMAT partial arc V20v	50.6 (42.9–58.4)	
			VMAT partial arc V40%	2.6 (1.0–4.2)	
			VMAT 1 arc V20%	30.1 (22.8–37.4)	
			VMAT 1 arc V40%	0.3 (-0.1 to 0.8)	
			VMAT 1 arc +partial arc V20%	46.8 (36.6–56.9)	
			VMAT 1 arc +partial arc V40%	0.4 (-0.3 - 0.1)	
			VMAT 2 arc V20%	30.4 (20.9–39.8)	
			VMAT 2 arc V40%	0.1 (-0.4 - 0.1)	
Onal et al. (2014) [83]	10/6 posterior	Right	IMRT 6MV D maximum	42.07 ± 4.18Gy	

			VMAT 6MV<br>D maximum	36.36 ± 7.11Gy	
			IMRT 10MV<br>D maximum	40.63 ± 4.02Gy	
			VMAT 10MV<br>D maximum	39.16 ± 6.69Gy	
			IMRT 15MV<br>D maximum	40.96 ± 4.38Gy	
			VMAT 15MV<br>D maximum	39.80 ± 6.59Gy	
		Left	IMRT 6MV<br>D maximum	40.98 ± 3.96Gy	
			VMAT 6MV<br>D maximum	40.33 ± 6.21Gy	
			IMRT 10MV<br>D maximum	39.90 ± 4.14Gy	

			VMAT 10MV<br>D maximum	40.33 ± 5.24Gy	
			IMRT 15MV<br>D maximum	40.14 ± 4.30Gy	
			VMAT 15MV<br>D maximum	40.42 ± 5.29Gy	
Scobiola et al. (2016) [86]	5/3 posterior	Right	Tomoterapy<br>D1cc	18.0 ± 2.9Gy	
			IMRT<br>D1cc	32.0 ± 6.2Gy	
			VMAT<br>D1cc	28.1 ± 5.3Gy	
			Proton therapy<br>D1cc	35.2 ± 3.9Gy	
		Left	Tomoterapy<br>D1cc	18.1 ± 2.9Gy	
			IMRT<br>D1cc	31.9 ± 5.1Gy	
			VMAT<br>D1cc	26.6 ± 5.0Gy	
			Proton therapy<br>D1cc	35.5 ± 2.5Gy	

Pokhler et al. 2021 [40]	3		Halcyon VMAT<br>D 10cc	13.03 ± 1.65	
			Truebeam VMAT<br>D 10cc	12.83 ± 1.35	
Bartlett et al. (2023) [89]	5	Right	IMRT<br>D maximum	52.16 ± 4.72Gy	
			VMAT<br>D maximum	50.83 ± 8.89Gy	
			VMAT partial arc<br>D maximum	47.50 ± 9.59Gy	
		Left	IMRT<br>D maximum	52.30 ± 3.53Gy	
			VMAT<br>D maximum	51.48± 5.23Gy	
			VMAT partial arc<br>D maximum	50.72 ± 6.13Gy	
Fathy et al. (2023) [90]	7/4 posterior	Right	VMAT FF<br>D maximum	39.3 ± 2.6Gy	
			VMAT FFF<br>D maximum	39.2 ± 2.7Gy	
			VMAT MLCi2	39.2 ± 3.1Gy	

			D maximum		
		Left	VMAT FF<br><br>D maximum	39 ± 2.1Gy	
			VMAT FFF<br><br>D maximum	39 ± 2.1Gy	
			VMAT MLCi2<br><br>D maximum	39.8 ± 2.8Gy	
**Sigmoid**					
Onal et al. (2014) [83]	10/6 posterior	IMRT 6MV<br><br>D maximum	26.34 ± 17.23Gy		
		VMAT 6MV<br><br>D maximum	24.85 ± 17.01Gy		
		IMRT 10MV<br><br>D maximum	26.00 ± 17.10Gy		
		VMAT 10MV<br><br>D maximum	26.16 ± 17.80Gy		
		IMRT 15MV<br><br>D maximum	27.15 ± 16.81Gy		
		VMAT 15MV<br><br>D maximum	26.68 ± 17.55Gy		
Bartlett et al. (2023) [89]	5	IMRT<br><br>D maximum	62.34 ± 12.92Gy		
		VMAT D maximum	62.06 ± 13.69Gy		
		VMAT<br><br>Partial arc D maximum	60.62 ± 13.36Gy		

**Penile bulb**				
Pokhler et al. (2021) [40] Penile bulb	3	D3cc	Halcyon VMAT 4.43 ± 4.03	
		D3cc	Truebeam VMAT 3.98 ± 3.44	
Fathy et al. (2023) [90]	7/4 posterior	VMAT FF D mean	23.3 ± 10 Gy	
		VMAT FFF D mean	22.7 ± 9.75 Gy	
		VMAT MLCi2 D mean	24.7 ± 8.3 Gy	
**Urethra**				
Pokhler et al. (2021) [40] Urethra	3	D0.03cc	Halcyon VMAT 37.26 ± 0.45	
		D0.03cc	Truebeam VMAT 37.29 ± 0.42	
Faccenda et al. (2023) [77]	2	D0.035 cc	+1.0% (-1.6–5.6)	
		D10%	+0.7% (-1.2–4.9)	
**Bowel Bag**				
Bartlett et al. (2023) [89]	5	IMRT V45Gy	33.3 ± 29.2	
		VMAT V45Gy	33.9 ± 26.2	
		VMAT Partial arc	32.6 ± 27.6	

		V45Gy		
Fathy et al. (2023) [90]	7/4 posterior	VMAT FF V45Gy	76.2 ± 53.1	
		VMAT FFF V45Gy	67.4 ± 59.2	
		VMAT MLCi2 V45Gy	57.9 ± 46.8	
**Illiac Crest**				
Bartlett et al. (2023) [89]	5	IMRT D mean	22.64± 3.48Gy	
		VMAT D mean	21.86 ±3.39Gy	
		VMAT Partial arc D mean	21.70± 3.40Gy	

Abbreviations: IMRT = intensity modulated radiation therapy; VMAT = volumetric modulated arc therapy; 3D CRT = three-dimensional conformal radiation therapy; mm = millimeters; MLCi = multileaf collimator; FF = flattening filter; FFF = flattening filter free; MV = megavoltage, Gy = Gray, CBCT = cone-beam computed tomography, CT = computed tomography, US = ultrasound, MRI = magnetic resonance imaging
